# Identification of the Integration/Excision Module and Regulatory Elements Involved in the Mobility of IME8, an Integrative and Mobilizable Element From Mosquitocidal *Lysinibacillus sphaericus*

**DOI:** 10.1111/1751-7915.70387

**Published:** 2026-05-24

**Authors:** Yifeng Hu, Ying Yang, Xiaomin Hu, Jacques Mahillon, Zubin Chen, Han Xia

**Affiliations:** ^1^ College of Life Sciences South‐Central Minzu University Wuhan Hubei China; ^2^ Laboratory of Food and Environmental Microbiology Université Catholique de Louvain Louvain‐la‐Neuve Walloon Brabant Belgium; ^3^ Hubei Jiangxia Laboratory Wuhan Hubei China; ^4^ Key Laboratory of Virology and Biosafety, Wuhan Institute of Virology Chinese Academy of Sciences Wuhan Hubei China

**Keywords:** horizontal gene transfer, integrative and mobilizable element (IME), *Lysinibacillus sphaericus*, mosquito control, mosquitocidal toxin

## Abstract

*Lysinibacillus sphaericus* , a bacterium successfully used in the control of mosquitoes, bears its insecticidal traits in GI8, a recently identified mosquitocidal genomic island. GI8 is renamed IME8 in the present work, as it displays a typical genetic organization of an Integrative and Mobilizable Element (IME) and its circularized form is not self‐conjugative but mobilizable by the pBsph‐like plasmid p1593. The IME8 integration module (*int*‐operon) encodes two integrase‐like proteins (Int1 and Int2) belonging to the family of tyrosine recombinases, and a hypothetical protein (Hp3). All three ORFs are necessary and function as an essential excision unit of IME8. The chimeric construct “*attL*‐*int1‐int2‐hp3‐kan‐attR*” (hereafter named mini‐IME8 cassette) displays integrating property. The integration is specific to an *acnL*‐*yolD*(*attB*)*‐uvrX* operon target region, which is not only distributed in various *L. sphaericus* isolates but is also present among other *Lysinibacillus* species. The regulation module, *reg*‐operon, encodes an HTH‐domain‐carrying protein (Reg16) and a putative lytic polysaccharide monooxygenase (LPMO17). Knockout of the *reg*‐operon remarkably increases IME8 excision and transcription levels of *int1*/*int2*/*hp3* compared to the wild‐type situation. However, expression of *reg16* or the complete *reg*‐operon both increase the *int*‐operon promoter (P_int_) activity in *β*‐galactosidase activity assays, suggesting a complex regulation of the *int*‐operon.

## Graphical abstract

Mosquitocidal genomic island IME8 carries an *int‐*operon essential for integration/excision and a *reg‐*operon activating the promoter P_int_ and repressing excision. Its integration target is present in *L. sphaericus* and beyond. IME8 and a mini‐IME8 cassette can be mobilized by a conjugative plasmid and display integrating properties into the specific target.

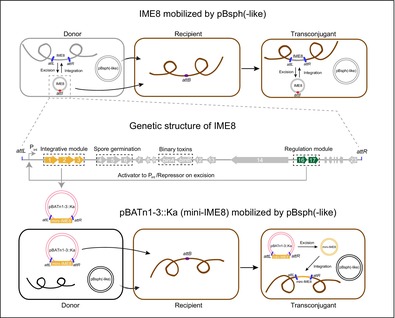

## 1 Introduction

Mobile Genetic Elements (MGEs) encompass a diverse array of genetic entities, including plasmids, bacteriophages, transposons, and the later‐defined Integrative and Conjugative Elements (ICEs). ICEs represent a distinct class characterized by their dual capability for conjugative transfer and genome integration, regardless of the specificity of their underlying molecular mechanisms (Frost et al. 2005). Structurally, ICEs consist of a combination of specialized functional modules that mediate excision/integration, horizontal transmission (mostly via Type IV Secretion System (T4SS)), stable maintenance within the host genome and coordinated regulation elements needed for these processes (Johnson and Grossman 2015). Integrative and Mobilizable Elements (IMEs) represent a recently discovered MGE class that differs from ICEs in their transfer mechanism. Unlike ICEs, IMEs cannot transfer independently but rely on co‐resident ICEs or conjugative plasmids within the donor host cell for their mobilization and transfer (Guédon et al. 2017; Parmeciano Di Noto et al. 2019). The transfer can occur either concurrently with the co‐transfer of the conjugative element, or independently by hijacking the conjugative machinery encoded by the coresident conjugative element without requiring its co‐transfer (Guédon et al. 2017; Parmeciano Di Noto et al. 2019). Unlike plasmids that have a sophisticated replication segregation system and are maintained in the host through autonomous replication, ICEs/IMEs integrate into a replicon of the host (plasmid or chromosome) via specific attachment sites called *attB* to ensure its vertical inheritance (Johnson and Grossman 2015; Guédon et al. 2017; Sun and Zhang 2023). The attachment site carried by circularized ICEs/IMEs is called *attI* (or *attP*). Following integration into *attB*, it produces typical or atypical inverted repeat sequences *attL* and *attR* flanking the ICE/IME element (Yang et al. 2022; Wang et al. 2024a). The ICEs/IMEs normally contain an integration module mainly consisting of recombinases (or referred to as integrase), that catalyse two recombination events (i. e., integration and excision) (Zakharova and Viktorov 2015). Most recombinases/integrases belong to either tyrosine or serine recombinase families (Smith and Thorpe 2002). Besides integrases, the recombination event may require directionality factor(s) (e. g., Xis and RDF), which can affect and favour recombination into a particular direction (excision vs. integration) (Khaleel et al. 2011). Many ICEs need particular physicochemical or environmental conditions to induce their excision or integration (Delavat et al. 2017; Armshaw and Pembroke 2016; Zhou et al. 2024; Nielsen and Hansen 2025). However, the regulation modules of ICEs/IMEs integration/excision are diverse and complex, and only in rare cases have their function been elucidated (Bi et al. 2012).

ICEs/IMEs mostly carry resistance genes, pathogenic factors or secondary metabolic genes, that play a huge driving role in the adaptive evolution of microorganisms and therefore have great research values (Durrant et al. 2020; Botelho and Schulenburg 2021). In addition, ICEs/IMEs can be genetically engineered for efficient and inducible DNA transfer to “undomesticated” bacteria Brophy et al. (2018). The number of reported ICEs/IMEs was estimated to be less than 10% of reported plasmids (Botelho and Schulenburg 2021), probably due to the difficulty in discovering ICEs/IMEs by wet experimental technologies. However, with the development and application of dry experimental technologies (high‐throughput sequencing and bioinformatic analysis), a variety of ICEs and IMEs have now been predicted, waiting for further characterization and mechanistic exploration.

*Lysinibacillus sphaericus* is an aerobic, Gram‐positive, spore forming bacterial species, for which some isolates display specific toxicity against mosquito larvae and have been successfully used worldwide for the control of mosquito‐borne diseases (e. g., malaria, dengue, filariasis or yellow fever) (Tangsongcharoen et al. 2015; Berry 2012). The genomes of the mosquitocidal isolates are highly conserved, and the toxin genes are mostly located on MGEs (Ge et al. 2011; Xu et al. 2015). Recently, we identified two types of MGEs in *L. sphaericus* : the conjugative plasmid pBsph and its pBsph‐like variants (e. g., p1593 and p2362) carrying a Type IV Secretion System (T4SS), and GI8 (renamed hereafter IME8), a *ca*. 30‐kb genomic island capable of integration and excision via recombination between its *attI* site and a specific *attB* region present on the chromosome or on pBsph(−like) plasmids (Geng et al. 2021). IME8 is not self‐conjugative but can be co‐transferred with pBsph(−like) plasmid as a composite element by integration into its *attB* site, then excising from pBsph and re‐integrated into the chromosomal *attB* site of the new host (Geng et al. 2021). Interestingly, the binary toxin genes *binA* and *binB* are located within IME8. Moreover, our recent study found that the two‐component toxin genes *cry48/tpp49* homologous to the three‐domain Cry toxins of *Bacillus thuringiensis* are also located on an ~35 kb IME8‐like genomic island (Chen and Hu et al. unpublished). It is hypothesized that the horizontal transmission of IME8 has somehow been associated to the evolution and ecological diversity of mosquito killing bacteria. We therefore propose that IME8 could be used as a novel genetic resource to construct engineered strains for accelerating the dissemination of mosquitocidal or other valuable traits in natural bacterial populations via HGT.

In order to evaluate the biotechnological potential of IME8 as a genetic engineering resource, the key factors related to the function of IME8 were identified in this study. The results showed that the mosquitocidal genomic island IME8 carries an *int‐*operon essential for integration/excision and a *reg‐*operon which can activate the promoter of the *int‐*operon and repress excision of IME8. Its integration target is present in *L. sphaericus* isolates and beyond. IME8 and a mini‐IME8 cassette carrying the chimeric construct “*attL*‐*int1‐int2‐hp3‐kan‐attR*” can be mobilized by a conjugative plasmid and display integrating property into the specific target. This work provides strong evidence that IME8 plays an important role in the evolution and dissemination of mosquitocidal traits within *Lysinibacillus* and establishes a foundation for further engineering of horizontal gene transfer systems to genetically modify bacteria.

## 2 Materials and Methods

### 2.1 Strains, Plasmids and Culture Conditions

The strains and plasmids used in this study are listed in Table S1. G725 is a pBsph‐cured derivative of *L. sphaericus* C3‐41. Strain 1593 is a wild‐type (WT) *L. sphaericus* isolate, closely related to C3‐41, that carries the pBsph‐like conjugative plasmid p1593. Both G725 and 1593 carry chromosomal IME8. KellenQ and NRS1693 are WT *L. sphaericus* isolates without IME8, whereas only the former carries the integration site *attB* on its chromosome (Geng et al. 2021). BMB171 is an acrystalliferous *B. thuringiensis* sv. *israelensis* strain commonly employed as an expression host for *Bacillus* spp. functional genes (Qin et al. 2022). The *L. sphaericus* strains were grown in Luria‐Bertani (LB) medium at 30°C for normal growth or 42°C for screening mutants of homologous recombination. *Escherichia coli* JM109, used as a bacterial host for gene cloning, was grown in LB broth at 37°C. The antibiotic concentrations (μg/ml) for screening were as follows: kanamycin (Kan), 50; spectinomycin (Spc), 200; ampicillin (Amp), 100; tetracycline (Tet), 5; erythromycin (Erm), 25; and trimethoprim (Tp), 50.

### 2.2 Construction of Knockout Mutants and Complemented Strains

The primers used in this study are listed in Table S2. To knock out *int1* (*orf1* ^IME8^, aka Bsph_3182) (Geng et al. 2021), upstream and downstream fragments of *int1* were amplified from *L. sphaericus* C3‐41 genome, and a kanamycin resistance gene (*kan*) was amplified from plasmid pXK15 (Ge et al. 2014). The three fragments were ligated into the linearized pRN5101 using the In‐Fusion HD Cloning kit (TaKaRa/Clontech) to generate plasmid pRN1. The same strategy was used to construct other recombinant plasmids (pRN2, pRN3, pRN16‐17, and pRN18‐23). All constructs were verified by PCR and DNA sequencing.

Before electroporation into the *L. sphaericus* G725 and 1593 cells, the plasmids were methylated using *Hae*III methyltransferase and S‐adenosylmethionine (SAM) (New England Biolabs, USA) to prevent degradation by the host restriction‐modification system, as previously described (Fu et al. 2017). Based on the temperature‐sensitive feature of pRN5101, double‐crossover mutants were selected by sub‐lethal culturing at 42°C (Poncet et al. 1997). Transformants resistant to kanamycin but sensitive to erythromycin were selected, and the mutants (e. g., G725Δ*int1*, 1593Δ*int1*, etc.) (Table S1) were confirmed by PCR and sequencing analysis.

For complementation, a fragment containing the *int*‐operon promoter and *attL* sequence (Geng et al. 2021) was amplified (primers P_int_‐F1/R1) and ligated into *Hin*d III/*Pst* I digested pBU4 to create pBAT. Then, *int1*, *int2*, *hp3*, and the entire *int*‐operon were amplified and ligated into *Pst* I/*Sal* I‐digested pBAT, yielding pBATn1, pBATn2, pBATn3, and pBATn1‐3. These plasmids were introduced into corresponding knockout strains to generate complemented strains (Tables S1 and S2).

Similarly, fragments containing *reg16*, *lpmo17*, or *reg16‐17* along with their promoters were amplified and cloned into *Hin*d III/*Pst* I digested pBU4, producing pBR16, pBR17, and pBR16‐17. These were transformed into 1593Δ*reg16‐17* to obtain the complemented strains 1593Δ*reg16‐17*_cm16, _cm17, and _cm16‐17 (Tables S1 and S2).

### 2.3 Horizontal Transmission and Stability Assay of IME8

The horizontal transmission capability of IME8 was evaluated by biparental mating experiments. G725Δ*reg16–17* and 1593Δ*reg16‐17* (Table S1), in which the *reg‐*operon of IME8 was replaced by a kanamycin gene (hereafter named IME8::Kan^R^), were used as donor strains, while G725Δ0498 was used as the recipient strain. The latter is a pBsph‐cured mutant of C3‐41, which contains a chromosomal IME8 and has its chromosomal lspC3‐41 restriction‐modification [R‐M] system replaced by a spectinomycin‐resistant marker (Geng et al. 2021).

Conjugation transfer was performed by “drop‐on‐drop” mating experiments on an LB agar plate without antibiotics as previously described (Hinnekens et al. 2019a). Each mating experiment was repeated three times, independently. The transconjugants were screened on Kan‐Spc LB plates and detected by PCR using the primer pairs Kan‐F/R and Spc‐F/R for kanamycin and spectinomycin genes, respectively. Primers tubz‐F/R, specific to the *tubZ* replicon, were designed to detect the co‐transfer of the conjugative plasmid p1593 (Ge et al. 2014). The transfer frequencies were calculated as the ratio of transconjugants to donor cells (T/D).

The stability of IME8::Kan^R^ and p1593 in the transconjugants was assessed through several successive subcultures (*ca*. 10–12 generations per 12‐h cycle) at a 1:1000 dilution on antibiotic‐free LB medium. About every 30 generations, the cultures were diluted and plated on LB. One hundred colonies of each transconjugant were transferred onto LB plates, with or without kanamycin. The occurrence of IME8::Kan^R^ and p1593 was indicated by detection of the kanamycin gene and *tubZ* replicon by PCR. The assays were performed in triplicate, starting from independent colonies.

### 2.4 Construction of the Recombinant Strain Carrying the “Mini‐IME” Cassette

The kanamycin resistance gene (*kan*) was PCR‐amplified from plasmid pXK15 (Ge et al. 2014) with the *attR* sequence (5′‐AATATAAAATTTATATTAAAT‐3′) (Geng et al. 2021) incorporated at the 5′‐end, then ligated into *Xba*I/*Bam*HI‐digested pBATn1‐3, generating pBATn1‐3::Kan. This construction contains the mini‐IME cassette, featuring “*attL*‐*int1‐int2‐hp3‐kan‐attR*” chimeric modular sequences.

Furthermore, a recombinant plasmid pXS‐attB was constructed for exogenous introduction of the *attB* target into acrystalliferous isolates *L. sphaericus* NRS1693 (Geng et al. 2021) and *B. thuringiensis* BMB171 (Qin et al. 2022). An *attB*‐containing fragment was amplified from C3‐41 chromosome, and the spectinomycin gene was amplified from G725Δ0498 (Geng et al. 2021), then both fragments were ligated with the *EcoRI‐digested* vector pXK10 (Ge et al. 2014).

### 2.5 Integration/Excision Analysis of IME8‐Derivatives and “Mini‐IME” Cassette

To monitor integration/excision of the IME8‐derivatives in WT, KO mutant, and complemented strains, PCR analysis with an equal amount of template (100 ng/10 μL reaction) was performed using primer pairs chro‐a/b and chro‐c/d to detect *attL* and *attR* sequences (integrated forms), respectively, and chro‐b/c to identify the *attI* sequence in excised (circularized) IME8‐derivatives, as previously described (Geng et al. 2021).

In addition, the pBATn1‐3::Kan plasmid was electroporated into *L. sphaericus* KellenQ, which harbours a native chromosomal *attB* site (Geng et al. 2021). Integration of the mini‐IME8 cassette into *attB* was verified using junction‐specific primers chro‐a/chro‐b’ and chro‐c’/chro‐d (Tables S1 and S2).

Furthermore, to verify the integration of the mini‐IME8 cassette into a heterologous host carrying exotic (introduced) *attB* site, plasmids pBATn1‐3::Ka and pXS‐attB were simultaneously electroporated into either *L. sphaericus* NRS1693 or *B. thuringiensis* BMB171 (Qin et al. 2022). Integration was detected using the primer pairs attB‐F/chro‐b’ and chro‐c’/attB‐R.

### 2.6 Genomic DNA and RNA Extraction, and Operon Validation

One mL of *L. sphaericus* cells grown in LB medium to an OD_595_ ~ 0.8 was used for genomic DNA and total RNA extraction. The former was obtained by purification using FastPure Bacteria DNA Isolation Mini Kit (Vazyme, China), and the latter via TRIzol reagent (Vazyme, China) according to the manufacturer's instructions. The PrimeScript RT reagent Kit with gDNA Eraser (Takara, Japan) was used to synthesize cDNA according to the manufacturer's instructions. The obtained gDNA, RNA, and cDNAs were subjected to PCR using the P1‐P24 primers, which were designed based on the sequences of the candidate int‐ and reg‐operons and their upstream and downstream neighbouring genes in IME8 (Figure S1 and Table S2).

### 2.7 Q‐PCR and RT‐qPCR

To determine the relative abundance of *attI* in *L. sphaericus* strains G725, 1593, and their derivatives which remain the IME8 excision capability, real‐time quantitative PCR (qPCR) was performed using the iQ2 real‐time PCR detection system (Bio‐Rad, USA). An *attI*‐containing fragment was first amplified from the C3‐41 chromosome using the chro‐b/c primer pair and subsequently ligated into the linearized pMD18T vector using the In‐Fusion HD Cloning kit (TaKaRa/Clontech) to generate the recombinant plasmid pMD18T‐attI.

For quantification, standard curves were established for both the reference gene *adk* (Ge et al. 2011) and the target *attI*. The primer pairs adk‐RT‐F/R and attI‐RT‐F6/R6 were used with serially diluted plasmid DNA templates (pXK10‐Bin‐Cadk for *adk* and pMD18T‐attI for *attI*, respectively). Genomic DNA extracted from *L. sphaericus* G725, 1593, and their derivatives was then subjected to qPCR analysis (100 ng/10 μL reaction) using Taq Pro Universal SYBR qPCR Master Mix (Vazyme, China) according to the manufacturer's protocol. The obtained cycle threshold (Ct) values were applied to the respective standard curves to calculate the copy numbers of *adk* (x_adk_) and *attI* (x_attI_). The relative copy number of *attI* was determined by the ratio x_attI_/x_adk_ (Tie et al. 2022), as presented in Table S3.

Furthermore, the transcription levels of *binA* and *binB* were assayed by Reverse Transcription quantitative PCR (RT‐qPCR) with the reference gene *rpoB* using the 2^−ΔΔCt^ method (Ge et al. 2014). Briefly, the recipient strain G725Δ0498 was used as the control group, and one randomly selected transconjugant strain (T4) was used as the experimental group. The Δ*Ct* of *bin* in both groups was obtained by subtracting the *Ct* of *rpoB* from the *Ct* of *bin*. Then the Δ*Ct*_Bin_ of T4 was subtracted from that of the control group to obtain the value of ΔΔ*Ct*_Bin_, and the relative content of *bin* was calculated as 2^−ΔΔCtBin^. When calculating the relative expression levels of *int1*, *int2*, and *hp3* in the mutant strain, the WT *L. sphaericus* 1593 was used as the control group, and the relative expression levels of *int1*, *int2*, and *hp3* were calculated using the above method.

### 2.8 Bioinformatics Analysis

Amino acid sequences of the candidate CDSs (i. e., *int1*, *int2*, *hp3, reg16*, and *lpmo17*) were subjected to PSI‐Blast (https://blast.ncbi.nlm.nih.gov/Blast.cgi) in the NCBI databases (nr/swissprot/pdb). The homologous sequences obtained, except those with too large difference in length, were aligned with MEGA X (MUSCLE) (Kumar et al. 2018). The aligned sequences were then used to construct phylogenetic trees using the Neighbour‐Joining method (Saitou and Nei 1987) and visualized with itol (https://itol.embl.de/). Molecular characterization of these CDSs was performed using Expasy (https://web.expasy.org/protparam/). Sequence alignment and identification of conserved amino acid motifs of the potential integrases encoded by Int1 and Int2 were performed by HHpred (Söding et al. 2005) (https://toolkit.tuebingen.mpg.de/tools/hhpred) and CDD (https://www.ncbi.nlm.nih.gov/Structure/cdd/wrpsb.cgi).

### 2.9 The *β*‐Galactosidase Activity Assays

The promoter region (3105025–3,105,244 nt, CP000817) of the *int*‐operon was amplified using primers P_int_‐F2/R2, which was subsequently ligated into *Sal* I/*Xba* I‐linearized pBU4, pBR16, pBR17, and pBR16‐17, yielding the constructs pBP_int_, pBR16‐P_int_, pBR17‐P_int_, and pBR16‐17‐P_int_. Additionally, a *lacZ*‐containing fragment was excised from *Bam*H I/*Kpn* I digested pHT304‐18 *lacZ* (Perchat et al. 2011) and inserted into the constructs to generate pB‐P_int_‐Z, pBR16‐P_int_‐Z, pBR17‐P_int_‐Z, and pBR16‐17‐P_int_‐Z (Tables S1 and S2). All recombinant plasmids were then transformed into BMB171 (Qin et al. 2022).

The *β*‐galactosidase activity assays were performed as previously described (Perchat et al. 2011) with minor modifications. Briefly, 200 μL of overnight‐cultured BMB171 cells were transferred to a 96‐well plate in triplicate, and the OD_595_ was measured. Then, 2 mL of BMB171 culture was pelleted, resuspended in 500 μL of Z buffer (0.06 M Na_2_HPO_4_, 0.04 M NaH_2_PO_4_, 0.01 M KCl, 1 mM MgSO_4_, 1 mM dithiothreitol), and lysed using a sonicator (10% power). After centrifugation (12,000 rpm, 10 min), 100 μL of the supernatant was mixed with 700 μL of Z buffer and 200 μL of 4 mg/mL 2‐nitrophenyl‐*β*‐D‐galactoside. The reaction was incubated at 37°C for 15 min and terminated by adding 500 μL of 1 M Na_2_CO_3_. The reaction mixture was transferred to a 96‐well plate, and the absorbance at A_420_ was recorded (Perchat et al. 2011). The *β‐galactosidase* activity of the bacterial culture is expressed in standard Miller Units. 1 Miller Unit = 1000 × [A_420_/(t × v × OD_595_)] (“T” 15 min, “V” 2 mL) (Klaips et al. 2020). Each assay was independently repeated at least three times.

### 2.10 Statistical Analysis

The data for the plots were presented as the mean ± standard error of three independent replicates. Statistical analysis and graphing were performed using GraphPad Prism (Version 9.0, GraphPad software, San Diego, CA, USA) and Origin 2024 (OriginLab, USA) software. For the measurement of *attI* content in cells, the data were first assessed for normality and lognormality. Subsequently, one‐way ANOVA followed by Duncan's test was applied. The mRNA levels of *binA*, *binB*, *int1*, *int2*, and *hp3* were quantified using the 2^−ΔΔCt^ method and analysed via two‐way ANOVA with an unpaired Student's *t*‐test, supplemented by post hoc Tukey's correction. The same statistical approach used for *attI* content analysis was also employed for the determination of *β*‐galactosidase activity in cells. Differences were considered statistically significant at *p* < 0.05 (*), *p* < 0.01 (**), *p* < 0.001 (***).

## 3 Results

### 3.1 Reanalysis of IME8 Genetic Organization Indicates a Typical IME Structure

**FIGURE 1:**
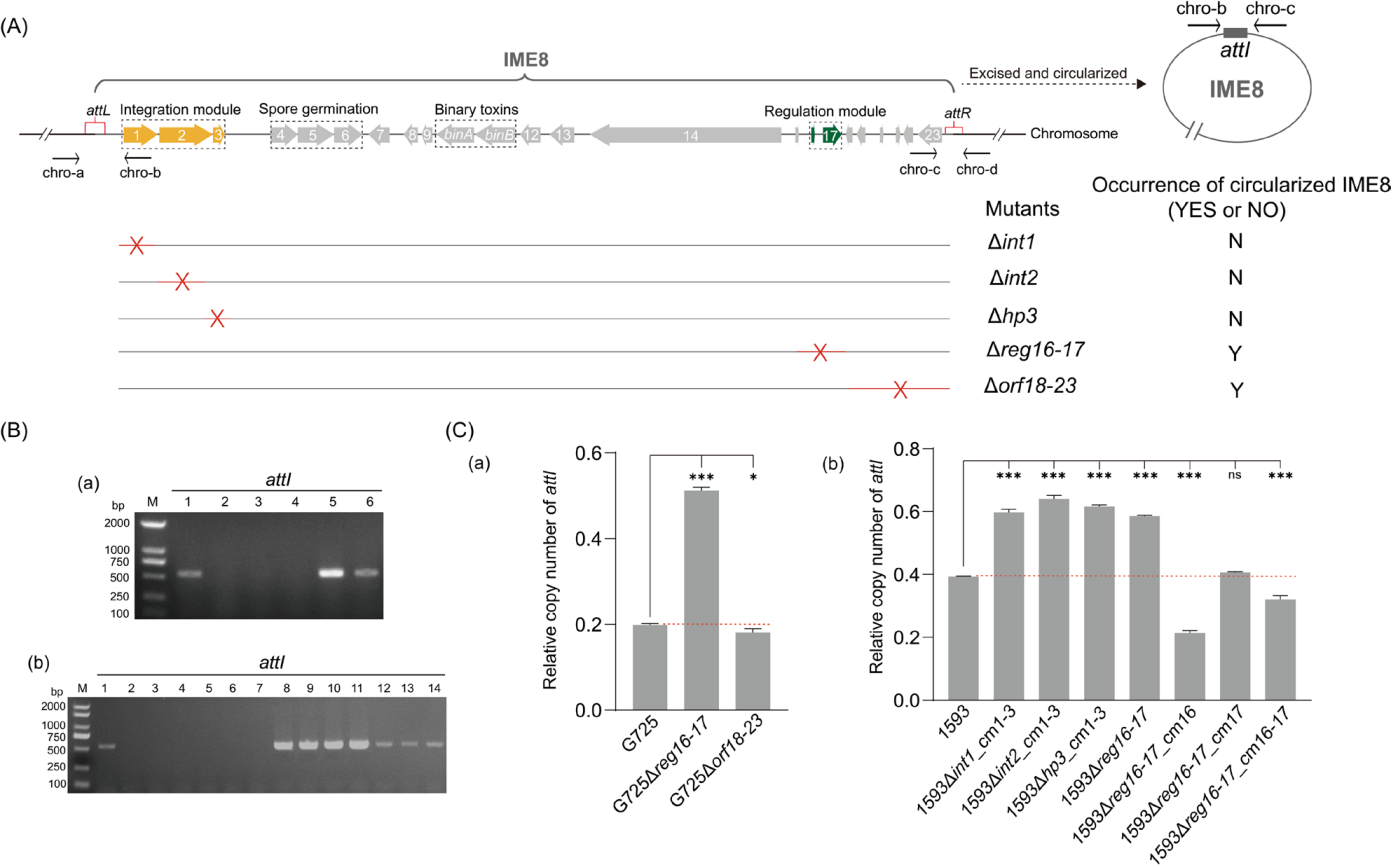
IME‐type genetic organization of IME8 (GI8). (A) Schematic diagram of IME8 (formerly GI8) functional modules, knockout strategy, and genomic integration/excision dynamics. The locations of primers chro‐a, chro‐b, chro‐c and chro‐d, used to detect the junction for integration and excision, are indicated by short black arrows. “Red crosses” refer to knocking out positions on IME8, while “Y or N” indicates that circularized IME8 can, or cannot, be detected in the mutants. The integration and regulation modules are marked in orange and blue, respectively. (B) PCR detection of IME8 excision in different mutants of *L. sphaericus* G725 (a) and 1593 (b). The primer pair chro‐b/chro‐c was used for detecting the new *attI* junction present in the circularized IME8. (a) Lanes 1–6: G725, G725Δ*int1*, G725Δ*int2*, G725Δ*hp3*, G725Δ*reg16–17*, G725Δ*orf18–23*. (b) Lanes 1–14: 1593, 1593Δ*int1*, 1593Δ*int2*, 1593Δ*hp3*, 1593Δ*int1*_cm1, 1593Δ*int2*_cm2, 1593Δ*hp3*_cm3, 1593Δ*int1*_cm1‐3, 1593Δ*int2*_cm1‐3, 1593Δ*hp3*_cm1‐31593Δ*reg16‐17*, 1593Δ*reg16‐17*_cm16, 1593Δ*reg16‐17*_cm17, 1593Δ*reg16‐17*_cm16‐17. M: DL 2000 DNA Marker. (C) Relative copy number of *attI* fragments in wild, knockout and complemented strains of *L. sphaericus* G725 (a) and 1593 (b). Only those that were *attI*‐positive by PCR were quantified by q‐PCR. An *attI*‐containing recombinant plasmid pMD18T‐attI and an *adk*‐containing recombinant plasmid pXK10‐Bin‐Cadk were used for qPCR analysis. The copy numbers of the reference gene *adk* (x_adk_) and *attI* (x_attI_) were calculated based on their Ct values and respective standard curves obtained by the qPCR analysis, while the relative copy number of *attI* was determined by the ratio x_attI_/x_adk_. *p < 0.05; ***p < 0.001; “ns” not significant.

**FIGURE 2:**
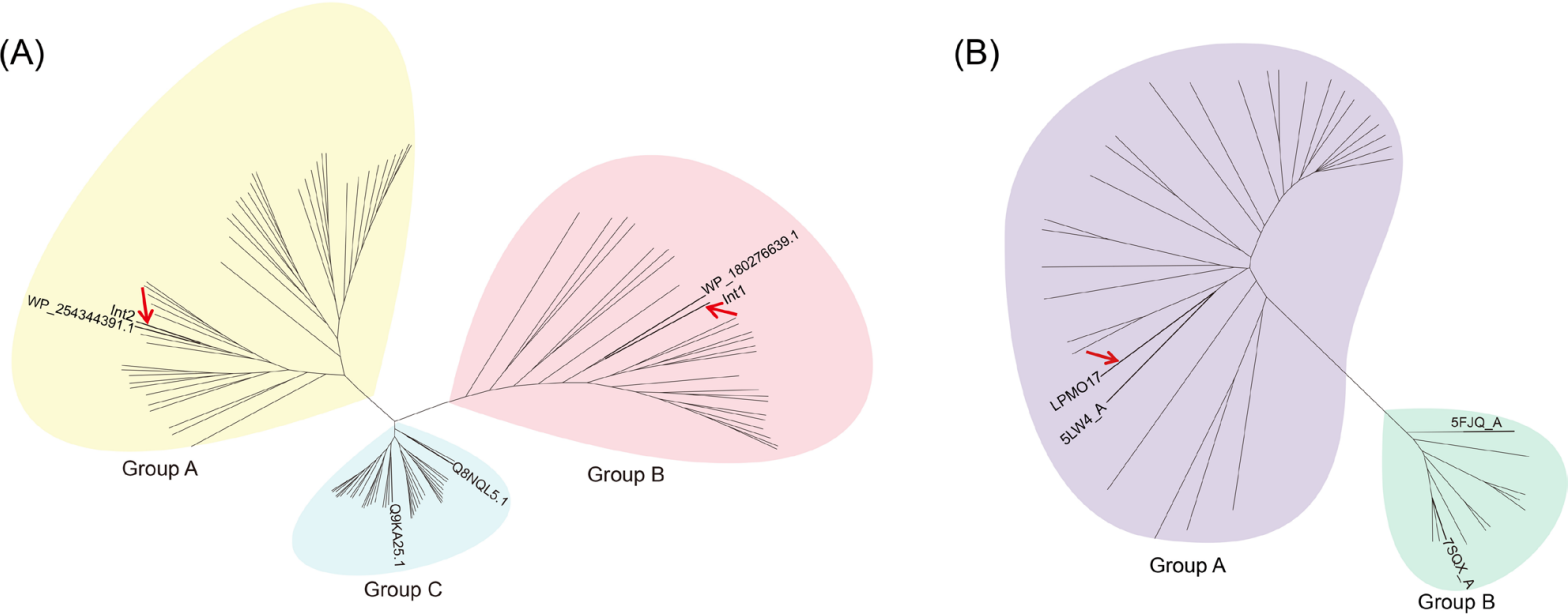
Phylogenetic analysis of Int1, Int2, and LPMO17. (A) Int1 is classified into Group A (yellow) and exhibits the highest identity (93%) to a predicted site‐specific integrase from *L. sphaericus* (WP_180276639.1), belonging to the tyrosine_recombinase_XerCD family. Int2 is classified into Group B (pink) and displays the highest identity (42%) to an integrase‐like protein encoded by *Paraburkholderia* sp. CNPSo 3274 (WP_254344391.1), belonging to the integrase‐like DNA breaking‐rejoining enzyme superfamily. In Group C (blue), Q8NQL5.1 (28.65% identity to Int1) from *Corynebacterium glutamicum* ATCC 13032 was predicted to encode a XerD‐like tyrosine recombinase, while Q9KA25.1 (24.91% identity to Int1) from *Alkalihalobacillus (Bacillus) halodurans* C‐125 was predicted to encode a XerC‐like tyrosine recombinase. (B) LPMO17 is classified into Group A (purple), displaying 55% identity to a lytic polysaccharide monooxygenase (5LW4_A from *Bacillus licheniformis* ). In Group B (green), 5FJQ_A (30.16% identity to LPMO17) from *Cellvibrio japonicus* was predicted to be a member of the GbpA family, and 7SQX_A (36.72% identity to LPMO17) from *Pseudomonas aeruginosa* PAO1 was predicted to encode chitin‐binding protein CbpD. Int1 (ORF1^IME8^), Int2 (ORF2^IME8^) and LPMO17 (ORF17^IME8^) are indicated by red arrows.

IME8 carries a total of 23 ORFs (Figure 1A). Neighbouring the *attL* site (see below), *orf1* ^IME8^ (*int1*) and *orf2* ^IME8^ (*int2*) are predicted to encode integrase(−like) proteins. The former exhibits the highest identity (93%) to a predicted site‐specific integrase (WP_180276639.1) from *L. sphaericus* , which belongs to the Tyrosine_recombinase_XerCD family, while the latter showed 42% identity to WP_254344391.1, an integrase‐like protein encoded by *Paraburkholderia* sp. CNPSo 3274, pertaining to a DNA breaking‐rejoining enzyme superfamily (Figure 2A). Int1 and Int2 contain the R‐E‐K‐H‐R‐Y‐Y and R‐E‐K‐H‐R‐Q‐Y motifs that deviate slightly from the canonical conserved R_I_‐E/D_I_‐K‐H_II_‐R_II_‐H/W_III_‐Y motif observed in most tyrosine recombinases, with the roman numerals corresponding to the three catalytic signatures (Table 1). ORF3^IME8^ (Hp3) was annotated as a hypothetical protein with no significant homologue in public databases. The three ORFs are clustered with only a 9‐bp space between *int1* and *int2*, and 44‐bp overlap between *int2* and *hp3*. Moreover, RT‐PCR experiments using *ad hoc* primers located in a region covering the *Bsph_3181* to *orf4* ^IME8^ flanking sequences showed that the *int1‐int2‐hp3* genes are co‐transcribed as a single unit (Figure S1A,B).

**TABLE 1:** Conserved residues in Int1, Int2 and other representative tyrosine recombinase superfamily proteins.

Protein	Accession numbers^a^							
The canonical motif^b^		Arg I RI	Glu/Asp I E/DI	Lys K	His II HII	Arg II RII	His/Trp III H/WIII	Tyr Y
Int1	ACA40693.1	R239	E242	K265	H344	R347	**Y378**	Y390
Int2	ACA40694.1	R401	E404	K438	H531	R534	**Q558**	Y567
XerD_ *Enterococcus faecalis*	6EMY_A	R145	E148	**V176**	H264	R267	H290	Y300
XerD_ *E. coli*	1A0P_A	R146	E149	K170	H242	R245	H268	Y277
INT_Cre_Punavirus P1	1XO0_A	R154	E157	**V185**	H270	R273	W296	Y305
XerD_*Thermoplasma acidophilum* DSM 1728	5HXY_A	R170	E173	K195	H263	R266	H289	Y298
XerD_*Helicobacter pylori* 26,695	5JK0_B	R214	E217	K240	H310	R313	H336	Y345
IntI4_*Vibrio cholerae* O1 biovar El Tor str. N16961	2A3V_C	R135	E138	K160	H267	R270	H293	Y302
XerA_*Pyrococcus abyssi* (archaea)	4A8E_A	R135	E138	K160	H226	R229	H252	Y261
Int_Peduovirus P2	5C6K_B	R149	E152	K172	H224	R227	H250	Y259
Int‐DNA_Enterobacteria phage lambda	1Z1B_B	R212	D215	K235	H308	R311	H333	Y342
Int_*Staphylococcus aureus* subsp. *aureus* USA300_TCH1516	3NKH_B	R70	E73	**T94**	H187	R190	H213	Y223
XerD_ *E. coli* O6:H1 (strain CFT073/ATCC 700928/UPEC)	5DCF_A	R39	E42	K63	H135	R138	H161	Y170
Int_*Sulfolobus* spindle‐shaped virus 1	3VCF_A	R39	E42	**G69**	**K106**	R109	**R132**	Y142

*Note:* Substitutions in the conserved residues of the tyrosine recombinase superfamily proteins, compared to the canonical R_I_‐E/D_I_‐K‐H_II_‐R_II_‐H/W_III_‐Y motif, are shaded and in bold.

^a^ Accession number in GenBank for Int 1 and Int 2, and in PDB database for other tyrosine recombinases.

^b^ Roman numerals correspond to the three catalytic signature motifs I, II and III.

ORF4^IME8^, ORF5^IME8^, and ORF6^IME8^ encode spore germination‐related proteins, with an organization similar to genetic structures reported in other *Bacillus* MGEs (e. g., the class II transposon Tn*XO1* located on the pXO1 plasmid from *Bacillus anthracis* ) (Van der Auwera and Mahillon 2005). The (*orf10‐orf11*)^IME8^ operon encodes the binary toxins BinA and BinB, while *orf13* ^IME8^ encodes the hypothetical Mtx2/3 toxin protein as previously reported (Hu et al. 2008). *Orf14* ^IME8^ encodes Gramicidin S synthetase, a Nonribosomal Peptide Synthetase (NRPS) belonging to AMP‐binding super family (Figure 1A).

Closer to the *attR* site, *orf15* ^IME8^ was predicted to encode a hypothetical protein but no transcription could be observed based on RT‐PCR analysis (Figure S1). ORF16^IME8^ (Reg16) was predicted to be a hypothetical protein carrying an HTH‐binding domain, often associated with DNA‐binding regulators (Aravind et al. 2005a). ORF17^IME8^ (LPMO17) contains an LPMO_AA10 domain and a transmembrane domain. To date, the exact function of the LPMO_AA10 domain remains poorly defined. The presence of a predicted transmembrane domain implies that LPMO17 may act as a sensor involved in environmental stress responses. Notably, its closest homologue is a lytic polysaccharide monooxygenase 55% identity to 5LW4_A from *Bacillus licheniformis* (Figure 2B), a protein implicated in microbial environmental adaptation, pathogenicity and biomass degradation (Vandhana et al. 2022). Interestingly, the *reg16* and *lpmo17* genes were also shown to be co‐transcribed as an operon (Figure S1B) and were hypothesized as a candidate regulation module based on the HTH‐binding domain of Reg16.

Thus, IME8 carries a potential integration/excision module *int1‐int2‐hp3* (named *int*‐operon), a putative regulatory module *reg16‐lpmo17* (*reg*‐operon), a spore germination‐related (*orf4‐orf6*)^IME8^ module, the binary toxin genes *binA/binB* (i. e., (*orf10‐orf11*)^IME8^) together with other accessory genes, but no conjugative or replicative elements. This organization qualifies IME8 as a potential Integrative and Mobilizable Element.

### 3.2 The *Int*‐ and *Reg*‐Operons Are Key Modules Involved in IME8 Excision

A series of KO mutants were generated through homologous recombination in the pBsph‐cured *L. sphaericus* G725, leading to the mutants G725Δ*int1*, G725Δ*int2*, G725Δ*hp3*, G725Δ*reg16–17*, and G725Δ*orf18–23*. Similarly, KO mutants were constructed in *L. sphaericus* 1593 (which harbours the pBsph‐like plasmid p1593) by introducing pRN‐series plasmids, yielding 1593Δ*int1*, 1593Δ*int2*, 1593Δ*int3*, and 1593Δ*reg16‐17* (Table S1). Unfortunately, no 1593Δ*orf18‐23* could be obtained. We hypothesize that the region spanning *orf18* to *orf23* encodes factors that interact with p1593, and that this interaction may account for the observed difficulties in achieving its knockout.

Complementation experiments were also designed by constructing the recombinant plasmids pBATn1, pBATn2, pBATn3, and pBATn1‐3, containing *int1*, *int2*, *hp3* and the whole *int*‐operon, respectively (see Material & Methods for details), which were transformed into the *L. sphaericus* 1593 KO mutants, yielding the complemented strains 1593Δ*int1*_cm1, 1593Δ*int2*_cm2, 1593Δ*hp3*_cm3, 1593Δ*int1*_cm1‐3, 1593Δ*int2*_cm1‐3, and 1593Δ*hp3*_cm1‐3 (Table S1).

The excision capability of IME8‐derived mutants was determined by detecting the circularized *attI* sequence generated by *attL* × *attR* recombination (Figure 1A), using PCR (Figure 1Ba,Bb, Table S2); those that were *attI*‐positive were then quantified by q‐PCR (Figure 1Ca,Cb, Table S3). Compared to the WT strain, no *attI* was detected in Δ*int1*, Δ*int2*, or Δ*int3* mutants of both G725 and 1593 strains (Figure 1Ba,Bb). Although individual KO of these genes did not affect the transcription of the remaining genes in the operon (Figure S1Ab), individual complementation of *int1*, *int2*, or *hp3* in the respective KO mutants failed to restore *attI* production. Only when the entire *int*‐operon was complemented using the high‐copy‐number plasmid pBU4 did the *attI* production significantly restore (Figure 1Bb,Cb). These results indicate that the complete *int*‐operon functions as an indispensable unit for excision.

In contrast, deletion of the *reg*‐operon in both G725 and 1593 significantly increased *attI* levels, as consistently shown by PCR (Figure 1Ba,Bb) and qPCR (Figure 1Ca,Cb, Table S3). Complementation with *reg16*, *lpmo17*, or both reduced *attI* copy numbers relative to the KO mutant (Figure 1Bb,Cb), suggesting that the *reg*‐operon acts as a negative regulator of IME8 excision. Additionally, the relative *attI* copy number in WT 1593 (0.4) was twice that in G725 (0.2) (Table S3), a difference that may be attributed to the presence of the conjugative plasmid p1593 in strain 1593, potentially enhancing IME8 excision. This assumption is supported by a previous observation that IME8 excision frequency is also higher in the C3‐41 strain than in its pBsph‐cured derivative, G725 (Geng et al. 2021). We therefore hypothesise that both conjugative plasmids, p1593 and pBsph, can mobilise IME8, likely by upregulating its excision from the chromosome to facilitate the subsequent conjugative transfer.

### 3.3 Excised/Circularized IME8::Kan^R^ Is Not Self‐Conjugative but Mobilizable by p1593

Considering that the KO of the *reg*‐operon in G725 and 1593 resulted in increased IME8 excision as indicated by the yield of circularized *attI*, G725Δ*reg16–17* and 1593Δreg16–17 were used as donor strains in mating‐out experiments, where G725Δ0498 was used as the recipient strain for the transmission of the IME8 in which the *reg‐*operon was replaced by a kanamycin gene (therefore named IME8::Kan^R^).

**FIGURE 3:**
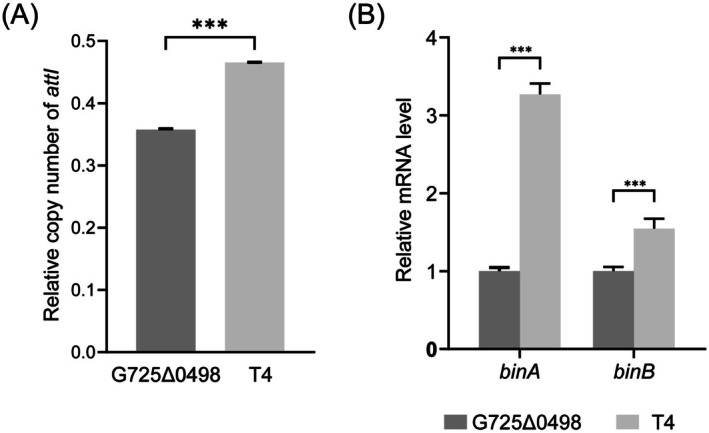
Relative copy number of *attI*, and relative mRNA levels of *binA* and *binB* in G725Δ0498 and the transconjugant T4. (A) The relative copy number of *attI* was determined by q‐PCR as the ratio of the copy number of *attI* (x_attI_) to that of the reference gene *adk* (x_adk_). (B) The transcription levels of *binA* and *binB* were assayed by RT‐qPCR with the reference gene *rpoB* using the 2^−ΔΔCt^ method. The donor 1593Δ*reg16‐17* carries IME8::Kan^R^, in which the *reg‐*operon was replaced by a kanamycin gene. The recipient strain G725Δ0498 (Spc^*R*^) is a pBsph‐cured mutant of C3‐41, which contains a chromosomal IME8. Strain T4 (Kan^R^Spc^*R*^) is one randomly picked transconjugant containing p1593 (pBsph‐like) and IME8::Kan^R^, both transferred from the donor strain 1593Δ*reg16‐17*. ***p < 0.001.

As shown in Table 2, no transconjugants were obtained using G725Δ*reg16–17* as donor, suggesting that IME8::Kan^R^ is not self‐conjugative. Yet, when 1593Δ*reg16‐17*, which contains the p1593 plasmid, was used as donor, a transfer frequency of IME8::Kan^R^ was estimated at 1.99 × 10^−6^ ± 1.52 × 10^−6^ T/D. Three independent transconjugants (T4, T5, T6) were randomly picked; remarkably, the occurrence of the p1593 replicon gene *tubZ* was observed in the transconjugants (Figure S2), suggesting that IME8::Kan^R^ might be mobilized by p1593. Interestingly, the relative copy number of *attI* (Figure 3A) and mRNA levels of the binary toxin genes *binA* and *binB* (Figure 3B) in the randomly picked transconjugant (T4) were higher than those observed in the recipient G725Δ0498. This observation can be explained by the fact that the recipient itself contains a chromosomal IME8 carrying *binA*/*binB*. Therefore, the acquisition of IME8::Kan^R^ from the donor is likely to increase the expression level of *binA*/*binB* and the copy number of circularized *attI*. However, stability assays revealed that neither p1593 (undetectable beyond 60 generations) nor IME8::Kan^R^ (retention rates of 41.6% at 60 generations and 4.2% at 120 generations) were stably maintained in the transconjugants (Table 2).

**TABLE 2:** Transfer frequency and stability of IME8Δ*reg16–17*.

Donor	Recipient	Transfer frequency (T/D)^a^	Occurrence (%) under non‐selective pressure in the transconjugants^b^					
			0 generation		60 generations		120 generations	
			*tubZ*	*kan*	*tubZ*	*kan*	*tubZ*	*kan*
G725Δ*reg16–17*	G725Δ0498	< 10^−9^	—	—	—	—	—	—
1593Δ*reg16‐17*	G725Δ0498	1.99 × 10^−6^ ± 1.52 × 10^−6^	100	100	0	41.6	0	4.2

^a^ Three independent mating experiments were performed. The transfer frequency was estimated using the ratio transconjugants/donor ± SE.

^b^ The occurrence of plasmid replicon gene *tubZ* and *kan* revealed by PCR was used to indicate the presence of p1593 and IME8Δ*reg16–17* (in which the *reg‐*operon of IME8 was replaced by a kanamycin gene), respectively.

### 3.4 The *Int*‐Operon Function as an Integration Device

**FIGURE 4:**
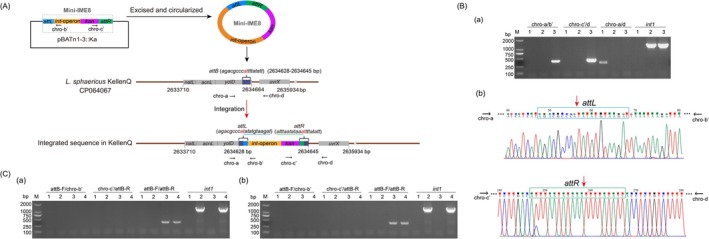
Integration analysis of the mini‐IME8 cassette. (A) Integration diagram of pBATn1‐3::Ka carrying mini‐IME8 cassette (i. e., “*attL*‐*int1‐int2‐hp3‐kan‐attR*” structure, named *int*‐operon) into the *attB* site of *L. sphaericus* KellenQ chromosome. Four predicted genes, *natL*, *acnL*, *yolD* and *uvrX* which are neighbouring the integration target *attB*, are indicated. (B) PCR detection (a) and sequencing (b) of the integration sites. Lanes 1–3 refer to KellenQ, pBATn1‐3::Ka and KellenQ (pBATn1‐3::Ka), respectively. Lane M: DL 2000 DNA Marker. The amplified fragments by the primer pairs chro‐a/b’ and chro‐c’/d were sequenced and found to be joint DNA fragments, of which one corresponds to the combination of the chromosome fragment of the recipient (match to 2,634,609–2,634,638, CP064067) and attL‐*int1* fragment (match to 3,105,107–3,105,409, CP000817) in the foreign plasmid pBATn1‐3::Ka, while the other corresponds to the combination of *attR* and the chromosome fragment of the recipient (match to 2,634,637–2,634,780, CP064067). (C) PCR detection of the integration of mini‐IME8 cassette into the introduced pXS‐attB in *L. sphaericus* NRS1693 (a) and BMB171 (b). Primer pair for attB‐F/chro‐b’ and chro‐c’/attB‐R were designed based on the sequences of pXS‐attB and mini‐IME8. (a) Lanes 1–4: NRS1693, pBATn1‐3::Ka, pXS‐attB, NRS1693 (pXS‐attB, pBATn1‐3::Ka). (b) Lanes 1–4: BMB171, pBATn1‐3::Ka, pXS‐attB, BMB171 (pXS‐attB, pBATn1‐3::Ka). M: DL 2000 DNA Marker.

In order to simulate the integration of IME8 into the *attB* target in WT *L. sphaericus* , a recombinant plasmid pBATn1‐3::Ka carrying the mini‐IME8 cassette was constructed (see Material & Methods for details). It was transformed into *L. sphaericus* KellenQ, which contains a chromosomal *attB* site, resulting in the recombinant strain KellenQ (pBATn1‐3::Ka) (Figure 4A). The primer pairs chro‐a/b’ and chro‐c’/d were used to capture the joint DNA sequences flanking the integration sites, of which one corresponds to the combination of the chromosome fragment of the recipient (match to 2,634,609–2,634,638, CP064067) and attL‐*int1* fragment (match to 3,105,107–3,105,409, CP000817) in the foreign plasmid pBATn1‐3::Ka, and the other corresponds to the combination of *attR* and the chromosome fragment of the recipient (match to 2,634,637–2,634,780, CP064067) (Figure 4Ba,Bb). These data confirmed the *bona fide* integration of the mini‐IME8 cassette into the *attB* site of *L. sphaericus* KellenQ chromosome, indicating its integrative capability.

Nevertheless, the integration of the mini‐IME8 cassette into the heterologous hosts *L. sphaericus* NRS1693 (Figure 4Ca) or *B. thuringiensis* BMB171 (Figure 4Cb) carrying introduced *attB* site failed to be detected. These observations suggest that the IME8 integration may be host‐specific and requires the cooperation of other gene(s) or factor(s) present in native *attB*‐carrying strains of *L. sphaericus* .

### 3.5 “*Acnl*‐*Yold*( *attB* )*‐uvrx*” Target Region Is Distributed Beyond *L. sphaericus*

**FIGURE 5:**
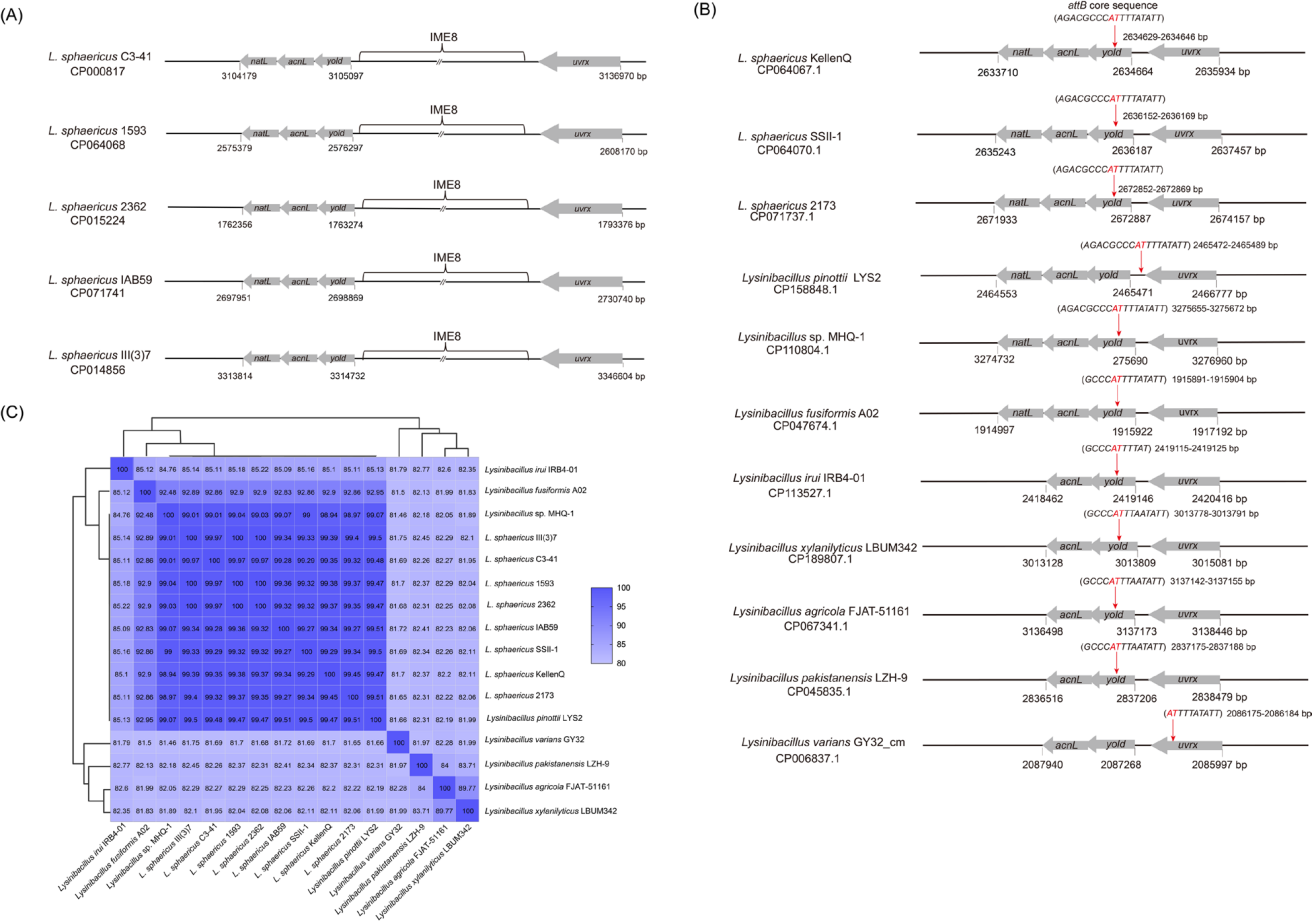
Occurrence of the *acnL*‐*yolD (attB)‐uvrX* target region among various *Lysinibacillus* spp. and related species. (A) Occurrence in toxic *L. sphaericus* with integrated IME8. (B) Occurrence in non‐toxic *L. sphaericus* and other *Lysinibacillus* isolates without integrated IME8. The *attB* sequences display variations compared with the reference *L. sphaericus* KellenQ. (C) Average Nucleotide Identity (ANI) analysis between *Lysinibacillus* spp. and related species carrying *acnL*‐*yolD‐uvrX*.

Within all the reported IME8‐carrying *L. sphaericus* isolates, e. g., C3‐41, 1593, 2362, IAB59, III(3)7, their integration *attB* site is located within *yolD*, which is flanked by the *acnL* and *uvrX* genes. Of note, this *acnL*‐*yolD*(*attB*)*‐uvrX* specific target consists of an operon associated with DNA damage repair (Permina et al. 2002). Remarkably also, this IME8 target region is not restricted to *L. sphaericus* but is also present in a variety of other *Lysinibacillus* species, e. g., *Lysinibacillus* sp. MHQ‐1, *L. pinottii* LYS2, *L. fusiformis* A02, *L. xylanilyticus* LBUM342, *L. irui* IRB4‐01, *L. pakistanensis* LZH‐9, *L. varians* GY32, in which the *attB* sequences and its locations display variations (Figure 5A).

Furthermore, the occurrence of an *acnL*‐*yolD‐uvrX* operon without *attB* was observed in *L. capsica* TSBLM and other phylogenetically related species such as *Psychrobacillus* sp. INOP01, *Solibacillus* sp. R5‐41, and *Metasolibacillus* sp. FSL H7‐0170 (data not shown). The isolates carrying identical *attB* target exhibit higher Average Nucleotide Identities (ANI) to the reference ( *L. sphaericus* C3‐41) than the isolates carrying mutated *attB* (Figure 5B,C).

### 3.6 *Reg16* and *Lpmo17* Function as Regulation Modules

**FIGURE 6:**
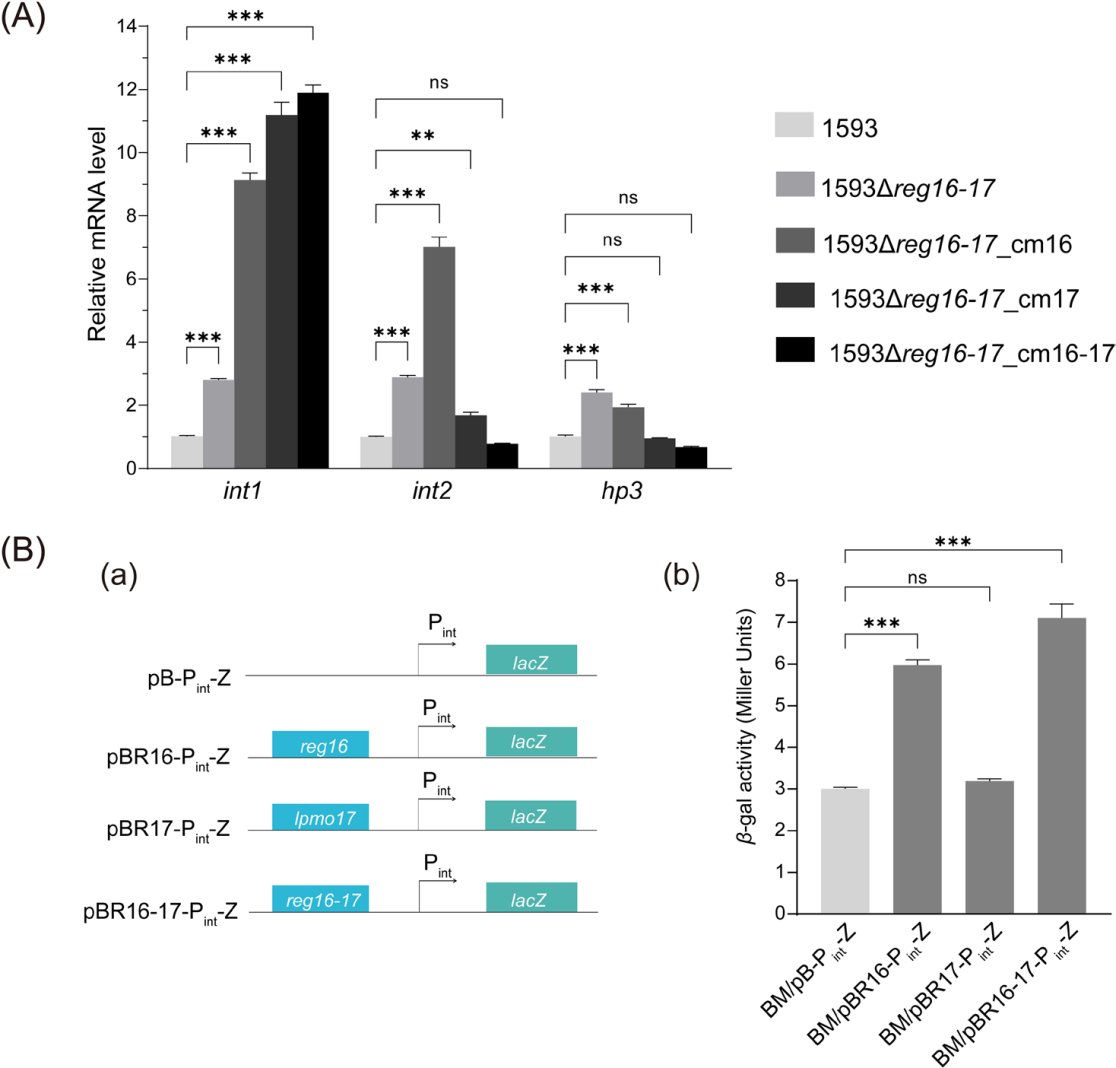
Regulation analysis of Reg16 and LPMO17. (A) Relative mRNA levels of *int1*, *int2*, and *hp3* in 1593Δ*reg16‐17* and complemented strains. (B) (a) Schematic diagram of the reporter plasmids and (b) their corresponding *β*‐galactosidase activities. **p < 0.01, ***p < 0.001, “ns” not significant.

Deletion of the whole *reg*‐operon led to significantly elevated transcription levels of *int1*, *int2*, and *hp3*, reaching approximately three‐fold that of WT strain 1593 (Figure 6A), suggesting a negative regulation by the *reg*‐operon. However, in the complemented strain 1593Δ*reg16‐17*_cm16, expression levels increased dramatically to nine‐fold (*int1*), seven‐fold (*int2*), and only two‐fold (*hp3*) relative to 1593 (Figure 6A). Similarly, strains 1593Δ*reg16‐17*_cm17 and 1593Δ*reg16‐17*_cm16‐17 exhibited > 10‐fold upregulation of *int1* expression, although the *int2* and *hp3* expression levels remained comparable to WT levels (Figure 6A).

In order to get further insights into the regulatory relationship between *reg16*/*lpmo17* and the *int*‐operon, we constructed reporter plasmids pB‐P_int_‐Z, pBR16‐P_int_‐Z, pBR17‐P_int_‐Z, and pBR16‐17‐P_int_‐Z for *β*‐galactosidase activity assays in BMB171 (Figure 6Ba). These plasmids allowed monitoring of the promoter of the *int*‐operon (P_int_) activity under the control of *reg16*, *lpmo17*, or both genes. As shown in Figure 6Bb, the *β*‐galactosidase activity was significantly increased in strains carrying pBR16‐P_int_‐Z and pBR16‐17‐P_int_‐Z, while pBR17‐P_int_‐Z showed activity comparable to the empty vector control. These results show that Reg16 is likely involved as a positive transcriptional regulator but also suggest that the fine‐tuning of the *int*‐operon regulation also relies on complex interactions between some of the gene products of the *int‐* and *reg‐*operons.

## 4 Discussion

### 4.1 Integration/Excision Mechanism of the *Int‐*Operon

Tyrosine recombinases typically display a seven key residues motif: [(Arg, Glu/Asp)I, Lys, (His)II, (Arg, His/Trp)III, Tyr], where roman numerals correspond to the three catalytic signature motifs I, II and III (Gibb et al. 2010). Both Int1 and Int2 carry this motif, with the His/Trp residue involved in stabilizing the transition state (Wang et al. 2018) substituted by a Tyr (Y) and a Gln (Q) residue, respectively (Table 1). Furthermore, different from the canonical integration/excision module in most MGEs, which often appear in a couple of tyrosine recombinases (Grainge and Jayaram 1999), the IME8 integrative/excision module encodes an auxiliary, yet functionally necessary hypothetical protein (Hp3). This suggests that the Int1‐Int2‐Hp3 module uses a distinct mechanism during excision/integration, potentially reflecting functional divergence within the tyrosine recombinase family. Large‐scale genomic analyses have revealed that IMEs commonly exhibit diverse modular recombination systems, often employing auxiliary genes to support excision and circularization (Lee et al. 2024). The three‐gene *int*‐operon of IME8 further illustrates such modular plasticity.

Remarkably, although single‐gene complementation in the KO mutants proved insufficient to restore production of excised IME8, complementation and overexpression of the full *int*‐operon using a high‐copy vector effectively rescued and enhanced its yield (Figure 1, Table S3). These findings strongly suggest that all three genes within the operon are indispensable and function cooperatively to facilitate the production of circularized IME8.

The mini‐IME8 cassette carrying the chimeric construct “*attL*‐*int1‐int2‐hp3‐kan‐attR*” displays integrating property into native *attB*‐carrying *L. sphaericus* but not into *B. thuringiensis* BMB171 with introduced *attB*‐carrying vector. Previous studies showed that despite the integrase and target site, some host‐specific cofactors are involved in the integration process. For instance, integration host factor (IHF) mediates site‐specific recombination and is indispensable for λ phage integration. It induces DNA bending, serving as a “molecular scaffold” that assembles integrase and other proteins at the correct site (Freundlich et al. 1992). The bacterial protein HU, which binds and bends DNA, may act as a substitute for or cooperate with IHF (Swinger and Rice 2007). The identity of the nucleoid‐associated proteins that mediate IME8 integration in *L. sphaericus* has yet to be elucidated.

### 4.2 Specific “*Acnl‐Yold*( *attB* )*‐uvrx”* Target Site of IME8

For many reported ICEs/IMEs, their *attB* target sites are usually located in tRNA gene (Menard and Grossman 2013). As documented in the ICEberg 3.0 database, numerous IMEs/ICEs target conserved non‐tRNA regions (Wang et al. 2024b). Consistent with this, the *attB* site of IME8 is located within *yolD*, which together with *acnL* and *uvrX* often belong to the *umuDC*‐like operon (Geng et al. 2021). Interestingly, it has been shown that the *umuDC* operon is in *Bacillus subtilis* prophages and *Enterococcus faecalis* transposons, functionally linked to polymerase V(−like) proteins which are regulated by the SOS repressor (Permina et al. 2002). It indicated that the specific integration into or excise from “*acnL‐yolD*(*attB*)‐*uvrX*” site of IME8 may also be related to or regulated by the SOS response. However, contrary to many MGEs that need to be induced by special chemical compounds (e. g., tetracycline for CTnDOT, 3‐chlorobenzoate for ICEclc) (Delavat et al. 2017; Zhou et al. 2024), by certain physicochemical (e. g., high cell density for ICEBs1, osmotic stress for IncI2, ethanol stress for MGE *412*) (Li and Zhang 2023; Vasil'eva et al. 2003) or environmental conditions (e. g., temperature and UV irradiation for ICE SXT/R391) (Armshaw and Pembroke 2016), no specific conditions triggering the integration and excision of IME8 have been detected so far (Geng et al. 2021). Nevertheless, in contrast to plasmid‐free isolates, a higher abundance of circularized IME8 was detected in isolates harbouring pBsph or p1593, suggesting that IME8 excision may be regulated by signals produced by conjugative plasmids. The region spanning *orf*18 to *orf*23 of IME8 could be knocked out in the plasmid‐free strain but not in the p1593‐carrying strain, suggesting that this region may include factors that interact with p1593, thereby complicating knockout efforts. Consistent with this observation, the intergenic region between *orf20* and *orf21* has been identified as the *oriT* site of IME8 (data not shown), which enables plasmid‐dependent mobilization.

Putative lytic polysaccharide monooxygenase (LPMOs) are generally recognized as extracellular enzymes that degrade recalcitrant polysaccharides (Courtade and Aachmann 2019). At present, only distant sequence similarity to characterized lytic polysaccharide monooxygenases can be identified, whereas the overall structural conservation and physiological function of this domain remain unclear. Moreover, structural prediction using AlphaFold3 and InterPro (data not shown) currently cannot provide definitive functional clues. There is still a lack of direct experimental evidence supporting its potential association with transcriptional regulation. Accordingly, the involvement of LPMO17 in modulating IME8 activity remains puzzling. Yet, the presence of a predicted transmembrane domain in LPMO17 suggests it might act as an environmental stress sensor. Nevertheless, the potential role of LPMO17 in regulating IME8 remains to be elucidated.

Remarkably, the *acnL‐yolD(attB)‐uvrX* target is not limited to *L. sphaericus* but also distributed in other species belonging to *Lysinibacillus*. It indicated that they might be potential recipients for IME8 and therefore become mosquitocidal. However, only a few isolates from other *Lysinibacillus* species have been found to display mosquitocidal features, e. g., *L. fusiformis* DSM 2898^T^ (Ahmed et al. 2007), *L. macroides* (Coorevits et al. 2012), *L. boronitolerans* 12B (Ahmed et al. 2007), *L. xylanilyticus* (Lee et al. 2010), and *L. mangiferihumi* (Dunlap 2019). Notably, some early reported mosquito‐killing *Lysinibacillus* strains were later reclassified as variants of *L. sphaericus* (Xu et al. 2015; Ahmed et al. 2007). Taking together, these observations provide strong evidence that IME8 is playing an important role in the spreading of the mosquitocidal features within *Lysinibacillus*.

### 4.3 Regulation Role of the *Reg*‐Operon

The regulation module of IME8 is the *reg*‐operon which likely acts as a repressor on excision. Deletion of the entire regulatory locus removed this repression and resulted in comparably increased transcription levels for all three *int*‐operon genes. Reg16 might serve as the primary transcriptional regulator of the *int*‐operon by regulating the operon promoter (P_int_) as indicated in *β*‐galactosidase activity assay. However, complementation/overexpression of *reg*16, *lpmo17*, or the complete *reg*‐operon yielded paradoxical results: all three manipulations led to a dramatic increase in the transcriptional level of *int1* while restoring that of *hp3*. Notably, complementation or overexpression of *lpmo17* alone specifically increased the transcription of *int2*. These observations may be attributable, among other things, to disrupted stoichiometry of the repressor components on unknown interactive mechanisms with Int1, Int2 and/or Hp3.

Similar phenomena have been reported for other bacterial repressors. For instance, the *E. coli* biotin repressor BirA binds its operator as a dimer, and cooperative interactions between subunits are essential for high‐affinity DNA binding; disruption of this stoichiometry impairs repression (Abbott and Beckett 1993). Similarly, the function of the central glycolytic gene repressor CggR is dependent on the precise stoichiometry of its subunits, and disruption of this stoichiometry leads to the loss of its repression (Chaix et al. 2010).

### 4.4 Ecological Implications and Biotechnological Potential for Vector Control

The low stability of IME8::Kan^R^ in the transconjugant population points towards a highly dynamic lifestyle, rather than a long‐term symbiotic relationship with the host. The rapid loss of p1593 in the new host as shown in Table 2 suggests its maintenance imposes a fitness cost. The slower loss of IME8::Kan^R^, even in its integrated state, indicates it either confers a burden or lacks positive selection. This dynamic mirrors a “hit‐and‐run” strategy of IME8 in *L. sphaericus* populations, where the primary goal is dissemination of the adaptation module (e. g., toxin genes). By transferring and subsequently being lost, IME8 spreads to new hosts while minimizing long‐term fitness costs. This “infective” lifestyle enables persistence through horizontal propagation rather than stable vertical inheritance, aligning with the general ecological strategy of IMEs (Tokuda and Shintani 2024).

Furthermore, IMEs represent versatile genetic tools for biotechnological exploitation and the engineering of beneficial traits in environmental bacteria (Tokuda and Shintani 2024). IMEs often carry large cargoes, enabling the insertion of complex metabolic pathways or multiple mosquitocidal genes. Engineering IME8 offers a strategy to introduce additional toxins into *L. sphaericus* carrying the target (e. g., KellenQ, 2173, Dak614) (Geng et al. 2021), potentially alleviating the resistance problem associated with the binary toxins BinA/BinB (Berry 2012). Moreover, it could facilitate the introduction of metabolic pathways that enable sugar utilization, thereby reducing fermentation costs—a current limitation of *L. sphaericus* , which lacks this capability (Berry 2012).

## 5 Conclusion

IME8, being a recently identified IME element, displays a typical IME genetic structure, carrying a *bona fide* integration/excision module, a regulation module, accessory genes such as toxin and NRPs antibiotic genes, but no conjugative and replicative element. IME8 transmission via mobilization by a conjugative plasmid promotes the transfer of mosquitocidal toxin genes. As a novel genetic resource, IME8 can be utilized in further studies to engineer HGT systems for the genetic modification of bacteria, accelerating the dissemination of mosquitocidal or other valuable traits in natural bacterial populations.

## Author Contributions

**Ying Yang:** investigation, writing – review and editing. **Yifeng Hu:** software, visualization, writing – original draft, data curation, investigation, writing – review and editing. **Zubin Chen:** investigation, writing – review and editing. **Han Xia:** investigation, writing – review and editing. **Xiaomin Hu:** conceptualization, methodology, resources, funding acquisition, supervision, writing – review and editing. **Jacques Mahillon:** writing – review and editing.

## Funding

This work was supported by National Natural Science Foundation of China, 32170008, 32211530564. key R&D Program of Hubei Jiangxia Laboratory, JXBS014, Fundamental Research Fund for the Central Universities of South‐Central Minzu University, CZZ26003.

## Ethics Statement

The authors have nothing to report.

## Conflicts of Interest

The authors declare no conflicts of interest.

## Supporting information


**Table S1:** Plasmids and bacterial strains used in this study.
**Table S2:** Primers used in this study.
**Table S3:**. Relative copy number of *attI* in wild type, knockout mutants and complemented strains of *L. sphaericus* .
**Figure S1:**. Validation of operons *int1‐3* (A) and *reg15‐17* (B).
**Figure S2:**. PCR detection of the transconjugants carrying circular IME8::Kan^R^ and p1593. The *reg16‐17* operon of the IME8 in the donor 1593Δ*reg16‐17* was replaced by a kanamycin gene and therefore named IME8::Kan^R^. The primer pair chro‐b/c was used for detecting the *attI* site witnessing the circular IME8::Kan^R^ and Tubz‐F/R was used for detecting the replicon gene of p1593. “ck”: wild‐type strain 1593, “D”: donor strain1593Δ*reg16–17*, “R”: recipient strain G725Δ0498. T4, T5, and T6 transconjugants were randomly picked after the mating experiments. M: DL 2000 DNA Marker. References to Supporting Information.

## Data Availability

The data that supports the findings of this study are available in the Supporting Information of this article.

## References

[mbt270387-bib-0001] Abbott, J. , and D. Beckett . 1993. “Cooperative Binding of the *Escherichia coli* Repressor of Biotin Biosynthesis to the Biotin Operator Sequence.” Biochemistry 32, no. 37: 9649–9656. 8373769 10.1021/bi00088a017

[mbt270387-bib-0002] Ahmed, I. , A. Yokota , A. Yamazoe , and T. Fujiwara . 2007. “Proposal of *Lysinibacillus boronitolerans* Gen. Nov. sp. Nov., and Transfer of *Bacillus fusiformis* to *Lysinibacillus fusiformis* Comb. Nov. and *Bacillus sphaericus* to *Lysinibacillus sphaericus* Comb. Nov.” International Journal of Systematic and Evolutionary Microbiology 57, no. 5: 1117–1125. 17473269 10.1099/ijs.0.63867-0

[mbt270387-bib-0003] Aravind, L. , V. Anantharaman , S. Balaji , et al. 2005a. “The Many Faces of the Helix‐Turn‐Helix Domain: Transcription Regulation and Beyond.” FEMS Microbiology Reviews 29, no. 2: 231–262. 15808743 10.1016/j.femsre.2004.12.008

[mbt270387-bib-0004] Armshaw, P. , and J. T. Pembroke . 2016. “UV stress‐responsive genes associated with enterobacterial integrative conjugative elements of the ICE SXT/R391 group. In Stress and Environmental Regulation of Gene Expression and Adaptation in Bacteria, UV Stress‐Responsive Genes Associated with Enterobacterial Integrative Conjugative Elements of the ICE SXT/R391 Group, Wiley”.

[mbt270387-bib-0005] Berry, C. 2012. “The Bacterium, *Lysinibacillus sphaericus* , as an Insect Pathogen.” Journal of Invertebrate Pathology 109: 1–10. 22137877 10.1016/j.jip.2011.11.008

[mbt270387-bib-0006] Bi, D. , Z. Xu , E. M. Harrison , et al. 2012. “ICEberg: A Web‐Based Resource for Integrative and Conjugative Elements Found in Bacteria.” Nucleic Acids Research 40, no. Database issue: D621–D626. 22009673 10.1093/nar/gkr846 PMC3244999

[mbt270387-bib-0007] Botelho, J. , and H. Schulenburg . 2021. “The Role of Integrative and Conjugative Elements in Antibiotic Resistance Evolution.” Trends in Microbiology 29, no. 1: 8–18. 32536522 10.1016/j.tim.2020.05.011

[mbt270387-bib-0008] Brophy, J. A. N. , A. J. Triassi , B. L. Adams , et al. 2018. “Engineered Integrative and Conjugative Elements for Efficient and Inducible DNA Transfer to Undomesticated Bacteria.” Nature Microbiology 3, no. 9: 1043–1053. 10.1038/s41564-018-0216-5 30127494

[mbt270387-bib-0009] Chaix, D. , M. L. Ferguson , C. Atmanene , et al. 2010. “Physical Basis of the Inducer‐Dependent Cooperativity of the Central Glycolytic Genes Repressor/DNA Complex.” Nucleic Acids Research 38, no. 17: 5944–5957. 20462860 10.1093/nar/gkq334 PMC2943609

[mbt270387-bib-0010] Coorevits, A. , A. E. Dinsdale , J. Heyrman , et al. 2012. “ *Lysinibacillus macroides* Sp. Nov., Nom. Rev.” International Journal of Systematic and Evolutionary Microbiology 62, no. 5: 1121–1127. 21724959 10.1099/ijs.0.027995-0

[mbt270387-bib-0011] Courtade, G. , and F. L. Aachmann . 2019. “Chitin‐Active Lytic Polysaccharide, Monooxygenases.” Advances in Experimental Medicine and Biology 1142: 115–129. 31102244 10.1007/978-981-13-7318-3_6

[mbt270387-bib-0012] Delavat, F. , R. Miyazaki , N. Carraro , N. Pradervand , and J. R. van der Meer . 2017. “The Hidden Life of Integrative and Conjugative Elements.” FEMS Microbiology Reviews 41, no. 4: 512–537. 28369623 10.1093/femsre/fux008 PMC5812530

[mbt270387-bib-0013] Dunlap, C. A. 2019. “ *Lysinibacillus mangiferihumi*, *Lysinibacillus tabacifolii* and *Lysinibacillus varians* Are Later Heterotypic Synonyms of *Lysinibacillus sphaericus* .” International Journal of Systematic and Evolutionary Microbiology 69, no. 9: 2958–2962. 31310193 10.1099/ijsem.0.003577

[mbt270387-bib-0014] Durrant, M. G. , M. M. Li , B. A. Siranosian , S. B. Montgomery , and A. S. Bhatt . 2020. “A Bioinformatic Analysis of Integrative Mobile Genetic Elements Highlights Their Role in Bacterial Adaptation.” Cell Host & Microbe 27, no. 1: 140–153. 31862382 10.1016/j.chom.2019.10.022 PMC6952549

[mbt270387-bib-0015] Freundlich, M. , N. Ramani , E. Mathew , A. Sirko , and P. Tsui . 1992. “The Role of Integration Host Factor in Gene Expression in *Escherichia coli* .” Molecular Microbiology 6, no. 18: 2557–2563. 1447969 10.1111/j.1365-2958.1992.tb01432.x

[mbt270387-bib-0016] Frost, L. S. , R. Leplae , A. O. Summers , and A. Toussaint . 2005. “Mobile Genetic Elements: The Agents of Open Source Evolution.” Nature Reviews. Microbiology 3, no. 9: 722–732. 16138100 10.1038/nrmicro1235

[mbt270387-bib-0017] Fu, P. , Y. Ge , Y. Wu , N. Zhao , Z. Yuan , and X. Hu . 2017. “The LspC3‐41I Restriction‐Modification System Is the Major Determinant for Genetic Manipulations of *Lysinibacillus sphaericus* C3‐41.” BMC Microbiology 17, no. 1: 116. 28525986 10.1186/s12866-017-1014-6 PMC5437673

[mbt270387-bib-0018] Ge, Y. , X. Hu , N. Zhao , T. Shi , Q. Cai , and Z. Yuan . 2014. “A New *tubRZ* Operon Involved in the Maintenance of the *Bacillus sphaericus* Mosquitocidal Plasmid pBsph.” Microbiology 160, no. Pt 6: 1112–1124. 24728200 10.1099/mic.0.075465-0

[mbt270387-bib-0019] Ge, Y. , X. Hu , D. Zheng , Y. Wu , and Z. Yuan . 2011. “Allelic Diversity and Population Structure of *Bacillus sphaericus* as Revealed by Multilocus Sequence Typing.” Applied and Environmental Microbiology 77: 5553–5556. 21685170 10.1128/AEM.00207-11 PMC3147464

[mbt270387-bib-0020] Geng, P. L. , J. Cheng , Z. Yuan , et al. 2021. “Horizontal Transfer of Large Plasmid With Type IV Secretion System and Mosquitocidal Genomic Island With Excision and Integration Capabilities in *Lysinibacillus sphaericus* .” Environmental Microbiology 23: 5131–5146. 33728723 10.1111/1462-2920.15467

[mbt270387-bib-0021] Gibb, B. , K. Gupta , K. Ghosh , R. Sharp , J. Chen , and G. D. van Duyne . 2010. “Requirements for Catalysis in the Cre Recombinase Active Site.” Nucleic Acids Research 38, no. 17: 5817–5832. 20462863 10.1093/nar/gkq384 PMC2943603

[mbt270387-bib-0022] Grainge, I. , and M. Jayaram . 1999. “The Integrase Family of Recombinases: Organization and Function of the Active Site.” Molecular Microbiology 33, no. 3: 449–456. 10577069 10.1046/j.1365-2958.1999.01493.x

[mbt270387-bib-0023] Guédon, G. , V. Libante , C. Coluzzi , S. Payot , and N. Leblond‐Bourget . 2017. “The Obscure World of Integrative and Mobilizable Elements, Highly Widespread Elements That Pirate Bacterial Conjugative Systems.” Genes 8, no. 11: 337. 29165361 10.3390/genes8110337 PMC5704250

[mbt270387-bib-0024] Hinnekens, P. , K. M. Kone , N. Fayad , A. Leprince , and J. Mahillon . 2019a. “pXO16, the Large Conjugative Plasmid From *Bacillus thuringiensis* Serovar *Israelensis* Displays an Extended Host Spectrum.” Plasmid 102: 46–50. 30825469 10.1016/j.plasmid.2019.02.004

[mbt270387-bib-0025] Hu, X. , W. Fan , B. Han , et al. 2008. “Complete Genome Sequence of the Mosquitocidal Bacterium *Bacillus sphaericus* C3‐41 and Comparison With Those of Closely Related *Bacillus* Species.” Journal of Bacteriology 190, no. 8: 2892–2902. 18296527 10.1128/JB.01652-07 PMC2293248

[mbt270387-bib-0026] Johnson, C. M. , and A. D. Grossman . 2015. “Integrative and Conjugative Elements (ICEs): What They Do and How They Work.” Annual Review of Genetics 49: 577–601. 10.1146/annurev-genet-112414-055018 PMC5180612 26473380

[mbt270387-bib-0027] Khaleel, T. , E. Younger , A. R. McEwan , A. S. Varghese , and M. C. M. Smith . 2011. “A Phage Protein That Binds φC31 Integrase to Switch Its Directionality.” Molecular Microbiology 80, no. 6: 1450–1463. 21564337 10.1111/j.1365-2958.2011.07696.x

[mbt270387-bib-0028] Klaips, C. L. , M. H. M. Gropp , M. S. Hipp , and F. U. Hartl . 2020. “Sis1 Potentiates the Stress Response to Protein Aggregation and Elevated Temperature.” Nature Communications 11, no. 1: 6271. 10.1038/s41467-020-20000-x PMC7722728 33293525

[mbt270387-bib-0029] Kumar, S. , G. Stecher , M. Li , C. Knyaz , and K. Tamura . 2018. “Mega X: Molecular Evolutionary Genetics Analysis Across Computing Platforms.” Molecular Biology and Evolution 35, no. 6: 1547–1549. 29722887 10.1093/molbev/msy096 PMC5967553

[mbt270387-bib-0030] Lee, C. S. , Y. T. Jung , S. Park , T. K. Oh , and J. H. Yoon . 2010. “ *Lysinibacillus xylanilyticus* Sp. Nov., a Xylan‐Degrading Bacterium Isolated From Forest Humus.” International Journal of Systematic and Evolutionary Microbiology 60, no. Pt 2: 281–286. 19651743 10.1099/ijs.0.013367-0

[mbt270387-bib-0031] Lee, E. , E. Pruitt , S. Woods , et al. 2024. “Genomic Analysis of Conjugative and Chromosomally Integrated Mobile Genetic Elements in Oral Streptococci.” Applied and Environmental Microbiology 90: e13605‐24. 10.1128/aem.01360-24 PMC11497809 39254330

[mbt270387-bib-0032] Li, L. G. , and T. Zhang . 2023. “Plasmid‐Mediated Antibiotic Resistance Gene Transfer Under Environmental Stresses: Insights From Laboratory‐Based Studies.” Sci Total Environ 887: 163870. 37149187 10.1016/j.scitotenv.2023.163870

[mbt270387-bib-0033] Menard, K. L. , and A. D. Grossman . 2013. “Selective Pressures to Maintain Attachment Site Specificity of Integrative and Conjugative Elements.” PLoS Genetics 9, no. 7: e1003623. 23874222 10.1371/journal.pgen.1003623 PMC3715440

[mbt270387-bib-0034] Nielsen, T. K. , and L. H. Hansen . 2025. “Single‐Strain Mobilome Sequencing Quantifies Bacterial Genetic Response to Stress, Including Activity of IS Elements, Prophages, RNAs, and REPINs.” Plasmid 134: 102759. 40998036 10.1016/j.plasmid.2025.102759

[mbt270387-bib-0035] Parmeciano Di Noto, G. , A. Iriarte , M. S. Ramírez , et al. 2019. “ICE SXT vs. ICESh95: Co‐Existence of Integrative and Conjugative Elements and Competition for a New Host.” Scientific Reports 9, no. 1: 8045. 31142760 10.1038/s41598-019-44312-1 PMC6541609

[mbt270387-bib-0036] Perchat, S. , T. Dubois , S. Zouhir , et al. 2011. “A Cell‐Cell Communication System Regulates Protease Production During Sporulation in Bacteria of the *Bacillus cereus* Group.” Molecular Microbiology 82, no. 3: 619–633. 21958299 10.1111/j.1365-2958.2011.07839.x

[mbt270387-bib-0037] Permina, E. A. , A. A. Mironov , and M. S. Gelfand . 2002. “Damage‐Repair Error‐Prone Polymerases of Eubacteria: Association With Mobile Genome Elements.” Gene 293, no. 1–2: 133–140. 12137951 10.1016/s0378-1119(02)00701-1

[mbt270387-bib-0038] Poncet, S. , C. Bernard , E. Dervyn , J. Cayley , A. Klier , and G. Rapoport . 1997. “Improvement of *Bacillus sphaericus* Toxicity Against Dipteran Larvae by Integration, via Homologous Recombination, of the *Cry11A* Toxin Gene From *Bacillus thuringiensis* Subsp. *Israelensis* .” Applied and Environmental Microbiology 63, no. 11: 4413–4420. 9361428 10.1128/aem.63.11.4413-4420.1997 PMC168761

[mbt270387-bib-0039] Qin, J. , Z. Cao , X. Cai , et al. 2022. “NupR Responding to Multiple Signals Is a Nucleoside Permease Regulator in *Bacillus thuringiensis* BMB171.” Microbiol Spectr 10, no. 4: e0154322. 35862946 10.1128/spectrum.01543-22 PMC9430930

[mbt270387-bib-0040] Saitou, N. , and M. Nei . 1987. “The Neighbor‐Joining Method: A New Method for Reconstructing Phylogenetic Trees.” Molecular Biology and Evolution 4, no. 4: 406–425. 3447015 10.1093/oxfordjournals.molbev.a040454

[mbt270387-bib-0041] Smith, M. C. , and H. M. Thorpe . 2002. “Diversity in the Serine Recombinases.” Molecular Microbiology 44, no. 2: 299–307. 11972771 10.1046/j.1365-2958.2002.02891.x

[mbt270387-bib-0042] Söding, J. , A. Biegert , and A. N. Lupas . 2005. “The HHpred Interactive Server for Protein Homology Detection and Structure Prediction.” Nucleic Acids Research 33: W244–W248. 15980461 10.1093/nar/gki408 PMC1160169

[mbt270387-bib-0043] Sun, S. , and X. Zhang . 2023. “Genetic Characteristics and Integration Specificity of *Salmonella enterica* Temperate Phages.” Frontiers in Microbiology 14: 1199843. 37593543 10.3389/fmicb.2023.1199843 PMC10428622

[mbt270387-bib-0044] Swinger, K. K. , and P. A. Rice . 2007. “Structure‐Based Analysis of HU‐DNA Binding.” Journal of Molecular Biology 365, no. 4: 1005–1016. 17097674 10.1016/j.jmb.2006.10.024 PMC1945228

[mbt270387-bib-0045] Tangsongcharoen, C. , N. Chomanee , B. Promdonkoy , and P. Boonserm . 2015. “ *Lysinibacillus sphaericus* Binary Toxin Induces Apoptosis in Susceptible *Culex quinquefasciatus* Larvae.” Journal of Invertebrate Pathology 128: 57–63. 25958262 10.1016/j.jip.2015.04.008

[mbt270387-bib-0046] Tie, Y. Y. , J. Q. Wen , and J. Tian . 2022. “Identification and Copy Number of *As6G‐FFT* in Transgenic Tobacco Plant.” Fujian Journal of Agricultural Sciences 37, no. 5: 592–599.

[mbt270387-bib-0047] Tokuda, M. , and M. Shintani . 2024. “Microbial Evolution Through Horizontal Gene Transfer by Mobile Genetic Elements.” Microbial Biotechnology 17: e14408. 38226780 10.1111/1751-7915.14408 PMC10832538

[mbt270387-bib-0048] Van der Auwera, G. , and J. Mahillon . 2005. “Tn*XO1*, a Germination‐Associated Class II Transposon From *Bacillus anthracis* .” Plasmid 53, no. 3: 251–257. 15848228 10.1016/j.plasmid.2004.08.004

[mbt270387-bib-0049] Vandhana, T. M. , J. L. Reyre , D. Sushmaa , J. G. Berrin , B. Bissaro , and J. Madhuprakash . 2022. “On the Expansion of Biological Functions of Lytic Polysaccharide Monooxygenases.” New Phytologist 233, no. 6: 2380–2396. 34918344 10.1111/nph.17921

[mbt270387-bib-0050] Vasil'eva, L. A. , V. A. Ratner , O. V. Antonenko , L. A. Vasilyeva , E. D. Lopukhova , and E. V. Bubenshchikova . 2003. “Induction of MGE 412 Transposition in an Isogenic Strain of *Drosophila melanogaster* by Different Doses of Ethanol Fumes.” Genetika 39, no. 5: 717–720. 12838621

[mbt270387-bib-0051] Wang, H. , G. Xie , and J. Huang . 2024a. “Genome‐Based Characterization of a Novel Prophage of *Vibrio parahaemolyticus* , VPS05ph1, a Novel Member of *Peduoviridae* .” Virology 595: 110087. 38636362 10.1016/j.virol.2024.110087

[mbt270387-bib-0052] Wang, J. , Y. Liu , Y. Liu , et al. 2018. “A Novel Family of Tyrosine Integrases Encoded by the Temperate Pleolipovirus SNJ2.” Nucleic Acids Research 46, no. 5: 2521–2536. 29361162 10.1093/nar/gky005 PMC5861418

[mbt270387-bib-0053] Wang, M. , G. Liu , M. Liu , et al. 2024b. “ICEberg 3.0: Functional Categorization and Analysis of the Integrative and Conjugative Elements in Bacteria.” Nucleic Acids Research 52, no. D1: D732–D737. 37870467 10.1093/nar/gkad935 PMC10767825

[mbt270387-bib-0054] Xu, K. , Z. Yuan , S. Rayner , and X. Hu . 2015. “Genome Comparison Provides Molecular Insights Into the Phylogeny of the Reassigned New Genus *Lysinibacillus* .” BMC Genomics 16, no. 1: 140. 25888315 10.1186/s12864-015-1359-x PMC4363355

[mbt270387-bib-0055] Yang, G. , A. Lin , X. Wu , et al. 2022. “ *Geobacter*‐Associated Prophages Confer Beneficial Effect on Dissimilatory Reduction of Fe(III) Oxides.” Fundamental Research 4, no. 6: 1568–1575. 39734524 10.1016/j.fmre.2022.10.013 PMC11670727

[mbt270387-bib-0056] Zakharova, I. B. , and D. V. Viktorov . 2015. “Integrative Conjugative Elements (ICEs) of Microorganisms.” Molecular Genetics, Microbiology and Virology 30, no. 3: 114–123.

[mbt270387-bib-0057] Zhou, H. , Z. Lu , X. Liu , et al. 2024. “Environmentally Relevant Concentrations of Tetracycline Promote Horizontal Transfer of Antimicrobial Resistance Genes via Plasmid‐Mediated Conjugation.” Food 13, no. 11: 1787. 10.3390/foods13111787 PMC11171790 38891015

