# Supplementary material for: Identification of the Integration/Excision Module and Regulatory Elements Involved in the Mobility of IME8, an Integrative and Mobilizable Element From Mosquitocidal Lysinibacillus sphaericus
Source: Microb Biotechnol. 2026 May 24;19(5):e70387. doi: 10.1111/1751-7915.70387 (PMC13239906; doi:10.1111/1751-7915.70387)
Supplement: Supplementary file 1 — Table S1: Plasmids and bacterial strains used in this study. Table S2: Primers used in this study. Table S3:. Relative copy number of attI in wild type, knockout mutants and complemented strains of L. sphaericus . Figure S1:. Validation of operons int1‐3 (A) and reg15‐17 (B). Figure S2:. PCR detection of the transconjugants carrying circular IME8::KanR and p1593. The reg16‐17 operon of the IME8 in the donor 1593Δreg16‐17 was replaced by a kanamycin gene and therefore named IME8::KanR. The primer pair chro‐b/c was used for detecting the attI site witnessing the circular IME8::KanR and Tubz‐F/R was used for detecting the replicon gene of p1593. “ck”: wild‐type strain 1593, “D”: donor strain1593Δreg16–17, “R”: recipient strain G725Δ0498. T4, T5, and T6 transconjugants were randomly picked after the mating experiments. M: DL 2000 DNA Marker. References to Supporting Information. [file MBT2-19-e70387-s001.docx]

Table S1: Plasmids and bacterial strains used in this study.

| **Strain or plasmid** | **Description** | **Sources or references** |
| --- | --- | --- |
| ***Plasmid*** |  |  |
| pRN5101 | Thermosensitive plasmid, shuttle vector of *E. coli* and *L. sphaericus*, Erm^R^ | (1) |
| pRN1 | pRN5101 carrying kanamycin gene flanked by fragment of *orf1*^IME8^ (3105268-3105825 and 3106001-3106040*), Erm^R^ and Kan^R^ | This study |
| pRN2 | pRN5101 carrying kanamycin gene flanked by fragment of *orf2*^IME8^ (3106714-3107449 and 3107920-3108534*), Erm^R^ and Kan^R^ | This study |
| pRN3 | pRN5101 carrying kanamycin gene flanked by fragment of *orf3*^IME8^ (3108269-3108788 and 3109135-3109670*), Erm^R^ and Kan^R^ | This study |
| pRN4-14 | pRN5101 carrying kanamycin gene flanked by fragment of *orf4-14*^IME8^ (3110918-3111483 and 3128913-3129602*), Erm^R^ and Kan^R^ | This study |
| pRN16-17 | pRN5101 carrying kanamycin gene flanked by fragment of *orf16-17*^IME8^ (3131156-3132407 and 3132624-3133448*), Erm^R^ and Kan^R^ | This study |
| pRN18-23 | pRN5101 carrying kanamycin gene flanked by fragment of *orf18-23*^IME8^ (3131038-3132358 and 3134677-3135230*), Erm^R^ and Kan^R^ | This study |
| pBU4 | High-copy-number shuttle vector of *E. coli* and *L. sphaericus*, Amp^R^ and Tet^R^ | (2) |
| pBAT | pBU4 carrying the *int*-operon promoter fragment (3105025-3105244*) containing the *attL* sequence, Amp^R^ and Tet^R^ | This study |
| pBATn1 | pBU4 carrying the *int*-operon promoter fragment (3105025-3105244*) containing the *attL* sequence and *orf1*^IME8^ (3105245-3106609*) gene, Amp^R^ and Tet^R^ | This study |
| pBATn2 | pBU4 carrying the *int*-operon promoter fragment (3105025-3105244*) containing the *attL* sequence and *orf2*^IME8^ (3106618-3108765*) gene, Amp^R^ and Tet^R^ | This study |
| pBATn3 | pBU4 carrying the *int*-operon promoter fragment (3105025-3105244*) containing the *attL* sequence and *orf3*^IME8^ (3108721-3109222*) gene, Amp^R^ and Tet^R^ | This study |
| pBATn1-3 | pBU4 carrying the *int*-operon promoter fragment (3105025-3105244*) containing the *attL* sequence and (*orf1-orf3*)^IME8^ (3105245-3109222*) gene, Amp^R^ and Tet^R^ | This study |
| pBATn1-3::Ka | pBU4 carrying the *int*-operon promoter fragment (3105025-3105244*) containing the *attL* sequence, (*orf1-orf3*)^IME8^ (3105245-3109222*) genes, kanamycin gene and *attR* sequence, Amp^R^, Tet^R^ and Kan^R^ | This study |
| pBR16 | pBU4 carrying *orf16*^IME8^ (3130771-3132319*) genes, Amp^R^ and Tet^R^ | This study |
| pBR17 | pBU4 carrying *orf17*^IME8^ (Promoter fragment 3130771-3132079* of *orf16*^IME8^ and fragment 3132355-3133017* of *orf17*^IME8^) gene, Amp^R^ and Tet^R^ | This study |
| pBR16-17 | pBU4 carrying (*orf16-orf17*)^IME8^ (3130771-3133017*) genes, Amp^R^ and Tet^R^ | This study |
| pXK10 | pUC19 carrying kanamycin gene and a 2.3-kb replicon fragment of pBsph | (3) |
| pXK10-Bin-Cadk | pXK10-Bin with a 0.5-kb fragment of *adk* gene from C3-41 chromosome, Kan^R^ | (4) |
| pMD18T | High-copy-number vector of *E. coli*, Amp^R^ | Sinobiological, China |
| pMD18T-attI | pMD18T (simple) carried *attI*-containing fragment amplified from C3-41 chromosome using the primer pairs chro-b/c, Amp^R^ | This study |
| pXS-attB | pXK10 carried attB-containing fragment amplified from C3-41 chromosome using the primer pairs chro-a/d, and spectinomycin gene (*spc*) amplified from G725Δ0498, Spc^R^ | This study |
| pHT304-18’*lacZ* | pHT304-18 carrying the *lacZ* gene, Amp^R^ | (5) |
| pB-P_int_ | pBU4 carrying the *int*-operon promoter fragment (3105025-3105244*), Amp^R^ and Tet^R^ | This study |
| pBR16-P_int_ | pBU4 carrying the *int*-operon promoter fragment (3105025-3105244*) and R16 (3130771-3132319*) gene, Amp^R^ and Tet^R^ | This study |
| pBR17-P_int_ | pBU4 carrying the *int*-operon promoter fragment (3105025-3105244*) and R17 (3130771-3132079* and 3132355-3133017*) gene, Amp^R^ and Tet^R^ | This study |
| pBR16-17-P_int_ | pBU4 carrying the *int*-operon promoter fragment (3105025-3105244*), and R16-17 (3130771-3133017*) genes, Amp^R^ and Tet^R^ | This study |
| pB-P_int_ -Z | pBU4 carrying the *int*-operon promoter fragment (3105025-3105244*), and 3 kb *lacZ* fragment, Amp^R^ and Tet^R^ | This study |
| pBR16-P_int_-Z | pBU4 carrying the *int*-operon promoter fragment (3105025-3105244*), R16 (3130771-3132319*) gene, and *lacZ* fragment, Amp^R^ and Tet^R^ | This study |
| pBR17-P_int_-Z | pBU4 carrying the *int*-operon promoter fragment (3105025-3105244*), R17 (3130771-3132079* and 3132355-3133017*) gene, and *lacZ* fragment, Amp^R^ and Tet^R^ | This study |
| pBR16-17-P_int_ -Z | pBU4 carrying the *int*-operon promoter fragment (3105025-3105244*), R16-17 (3130771-3133017*) genes, and *lacZ* fragment, Tet^R^ | This study |
| ***E. coli*** |  |  |
| JM109 | Cloning host | Lab stock |
| ***L. sphaericus*** |  |  |
| C3-41 | Wild type strain bearing native pBsph | (6) |
| G725 | C3-41 mutant cured of pBsph | (3) |
| G725Δ0498 | G725 mutant cured of pBsph-0498, Spec^R^ | (7) |
| G725△*int1* | G725 mutant with kanamycin gene taking the place of a 156-bp fragment of *orf1*^IME8^ in IME8, Kan^R^ | This study |
| G725△*int2* | G725 mutant with kanamycin gene taking the place of a 553-bp fragment of *orf2*^IME8^ in IME8, Kan^R^ | This study |
| G725△*hp3* | G725 mutant with kanamycin gene taking the place of a 348-bp fragment of *orf3*^IME8^ in IME8, Kan^R^ | This study |
| G725△*reg16-17* | G725 mutant with kanamycin gene taking the place of a 1147-bp fragment of *orf16-orf17*^IME8^ in IME8, Kan^R^ | This study |
| G725△*orf18-23* | G725 mutant with kanamycin gene taking the place of a 1501-bp fragment of *orf18-23*^IME8^ in IME8, Kan^R^ | This study |
| 1593 | Wild type strain bearing native p1593 which is a pBsph-like plasmid | (7) |
| 1593△*int1* | 1593 mutant with kanamycin gene taking the place of a 156-bp fragment of *orf1*^IME8^ in IME8, Kan^R^ | This study |
| 1593△*int2* | 1593 mutant with kanamycin gene taking the place of a 553-bp fragment of *orf2*^IME8^ in IME8, Kan^R^ | This study |
| 1593△*hp3* | 1593 mutant with kanamycin gene taking the place of a 348-bp fragment of *orf3*^IME8^ in IME8, Kan^R^ | This study |
| 1593△*reg16-17* | 1593 mutant with kanamycin gene taking the place of a 1147-bp fragment of (*orf16-orf17*)^IME8^ in IME8, Kan^R^ | This study |
| 1593△*int1*_cm1 | 1593△*int1* carrying pBATn1, Kan^R^ and Tet^R^ | This study |
| 1593△*int2*_cm2 | 1593△*int2* carrying pBATn2, Kan^R^ and Tet^R^ | This study |
| 1593△*hp3*_cm3 | 1593△*hp3* carrying pBATn3, Kan^R^ and Tet^R^ | This study |
| 1593△*int1*_cm1-3 | 1593△*int1* carrying pBATn1-3, Kan^R^ and Tet^R^ | This study |
| 1593△*int2*_cm1-3 | 1593△*int2* carrying pBATn1-3, Kan^R^ and Tet^R^ | This study |
| 1593△*hp3*_cm1-3 | 1593△*hp3* carrying pBATn1-3, Kan^R^ and Tet^R^ | This study |
| 1593△*reg16-17*_cm16 | 1593△*reg16-17* carrying pBR16, Tet^R^ | This study |
| 1593△*reg16-17*_cm17 | 1593△*reg16-17* carrying pBR17, Tet^R^ | This study |
| 1593△*reg16-17*_cm16-17 | 1593△*reg16-17*carrying pBR16-17, Tet^R^ | This study |
| KellenQ | Wild type strain without IME8 and plasmid whereas carrying *attB* on the chromosome | (7) |
| KellenQ (pBATn1-3::Ka) | KellenQ carrying pBATn1-3::Ka, Kan^R^ | This study |
| NRS1693 (pBATn1-3::Ka, pXS-attB) | NRS1693 carrying pBATn1-3::Ka and pXS-attB, Kan^R^ and Spc^R^ | This study |
| ***B. thuringiensis*** |  |  |
| BMB171 | Acrystalliferous mutant of *Bacillus thuringiensis* subsp. *israelensis* | (8) |
| BMB171 (pBATn1-3::Ka, pXS-attB) | BMB171 carrying pBATn1-3::Ka and pXS-attB, Kan^R^ and Spc^R^ | This study |
| BM/pB-P_int_-Z | BMB171 carrying pBP_int_-Z, Tet^R^ | This study |
| BM/pBR16-P_int_-Z | BMB171 carrying pBR16-P_int_-Z, Tet^R^ | This study |
| BM/pBR17-P_int_-Z | BMB171 carrying pBR17-P_int_-Z, Tet^R^ | This study |
| BM/pBR16-17-P_int_-Z | BMB171 carrying pBR16-17-P_int_-Z, Tet^R^ | This study |

*Based on the sequence of C3-41 chromosome (Accession No. CP000817)

Table S2 Primers used in this study.

| **Primer** | **Sequence (5’to 3’) ^a^** |  |
| --- | --- | --- |
| int1-LA-F | atggcgtgctgctagccttcgtcctgaagctcttgg | |
| int1-LA-R | cacctcaaatggttcggaatccatgtatcacccaca | |
| Kan-int1-F | tgatacatggattccgaaccatttgaggtgatagg | |
| Kan-int1-R | gtactgatggtcttcggtactaaaacaattcatccag | |
| int1-RA-F | aattgttttagtaccgaagaccatcagtacgcgaaa | |
| int1-RA-R | aagggcatcggtcgacgggtctttgatagggggttc | |
| int2-LA-F | atggcgtgctgctagctcgactggggattcgtacat | |
| int2-LA-R | cacctcaaatggttctgggcgatcccatattctaa | |
| Kan-int2-F | atatgggatcgcccagaaccatttgaggtgatagg | |
| Kan-int2-R | atctctgccttttggggtactaaaacaattcatccag | |
| int2-RA-F | aattgttttagtaccccaaaaggcagagatcaacc | |
| int2-RA-R | aagggcatcggtcgaccacaaccaacgcattgaaac | |
| hp3-LA-F | atggcgtgctgctagcttgtgggctcttggttacatc | |
| hp3-LA-R | cacctcaaatggttctgtttttctaaccatggtcgttc | |
| Kan-hp3-F | atggttagaaaaacagaaccatttgaggtgatagg | |
| Kan-hp3-R | ctgcgccaattaaacggtactaaaacaattcatccag | |
| hp3-RA-F | aattgttttagtaccgtttaattggcgcagaggaa | |
| hp3-RA-R | aagggcatcggtcgaccacgaaacgaattaaggcaaa | |
| orf4-LA-F | atggcgtgctgctagctcaagcagatggcgtttgta | |
| orf4-LA-R | cacctcaaatggttctaagcccttttagggaacca | |
| Kan-orf4-F | ccctaaaagggcttagaaccatttgaggtgatagg | |
| Kan-orf14-R | aatgcatatgggccaggtactaaaacaattcatccag | |
| orf14-RA-F | aattgttttagtacctggcccatatgcatttacaa | |
| orf14-RA-R | aagggcatcggtcgacttgtttccatgattggcgta | |
| reg16-17-LA-F | atggcgtgctgctagcaaagtgcttcgtccatccac | |
| reg16-17-LA-R | cacctcaaatggttccagtttttacgcacgttctca | |
| Kan-reg16-17-F | cgtgcgtaaaaactggaaccatttgaggtgatagg | |
| Kan-reg16-17-R | cccaaacggctagaaggtactaaaacaattcatccag | |
| reg16-17-RA-F | aattgttttagtaccttctagccgtttgggatgtc | |
| reg16-17-RA-R | aagggcatcggtcgactgttgcggaaggaaaatagg | |
| orf18-LA-F | atggcgtgctgctagcggcctttttggtggtatcct | |
| orf18-LA-R | cacctcaaatggttcggatggggaatcgaattttt | |
| Kan-orf18-F | ttcgattccccatccgaaccatttgaggtgatagg | |
| Kan-orf23-R | acctcgcagactgtcggtactaaaacaattcatccag | |
| orf23-RA-F | aattgttttagtaccgacagtctgcgaggtgacaa | |
| orf23-RA-R | aagggcatcggtcgacaaatccaagacgcgaaaatg | |
| P_int_-F1 | cccaagcttcgccaagcttatttccttgg | |
| P_int_-R1 | aaactgcaggaaatgattatgctcccttcaaaa | |
| int1-F | cccctgcagatgggcggtaaatatcactataagcttcg | |
| int1-R | cccgtcgacttcatacatttagctactccagtcc | |
| int2-F | cccctgcagatgaacattctagaacaatctaattcagaa | |
| int2-R | cccgtcgacttatttaattggtttgattctgacttctgg | |
| hp3-F | cccctgcagatgaaaactataaaccagaag | |
| hp3-R | cccgtcgactccccgaacattttagttgg | |
| P_reg16_-F | cccaagcttgtaaaggagatattcatttggtcccaat | |
| P_reg16_-R | atttttacctccaatttagcataagtaataaagtgcag | |
| reg16-R | cccgtcgactcatttacggtatttctgtaccgtacc | |
| lpmo17-F | gctaaattggaggtaaaaatatgatgaacactaaattctcaaaattaagc | |
| lpmo17-R | cccgtcgacttaaccatttggttgagatggg | |
| chro-a | tctgtcagctctctcggttg | |
| chro-b | tccttcccaagagcttcagg | |
| chro-c | gttcctgttactagccccgt | |
| chro-d | gctggatgagtactttgcgg | |
| TubZ-F | tggaacaaaaactaagttta | |
| TubZ-R | aagtaactttaataattct | |
| Kan-F | cgatcagggaggatatcgg | |
| Kan-R | gatcaagcctgattgggagaa | |
| Spc-F | gacggccagtgaattctacggggtctgacgctcagt | |
| Spc-R | ggacgggaatcattccctaggccatgggttttaaaag | |
| ERM-F | tttcttgtattctttgttaacccat | |
| ERM-R | tttgaaatcggctcaggaaaaggcc | |
| attI-RT-F6 | ctcccttcaaaattatta | |
| attI-RT-R6 | atagagtgacttgcaaaa | |
| adk-RT-F | gttgcgaaccgtctggaag | |
| adk-RT-R | gctgcggctgctctgtaat | |
| P1 | gtatctcatccagttcccat | |
| P2 | cctaattcatattggtaaaactcag | |
| P3 | gggcggtaaatatcactataag | |
| P4 | cgggtctttgatagggg | |
| P5 | gacaaaaataaattagggtttactgaa | |
| P6 | ccattaattcaatctcatgtagttc | |
| P7 | gaacattctagaacaatctaattcag | |
| P8 | cttacaaggaggtcaacac | |
| P9 | cctactccttctcaagttgc | |
| P10 | gggtaatctataaattgtttatacagcc | |
| P11 | gagaaatagaacgattgaattagtc | |
| P12 | ctccattgctcgttaatagg | |
| P13 | attgtcgaaaacaaccaaaa | |
| P14 | catcagagattggttttcgc | |
| P15 | gacggatgcatcaaagataa | |
| P16 | tcaggggtaatttacttctttcat | |
| P17 | gtgagaacgtgcgtaaaaac | |
| P18 | catttacggtatttctgtaccg | |
| P19 | ctgcactttattacttatgctaaattgg | |
| P20 | tgagttaaattagtcgatgcagc | |
| P21 | gcatttaaagggtctctcca | |
| P22 | ggagatgatggatgatgaacact | |
| P23 | gaagtaacttttgttgtatgttaacca | |
| P24 | cgctctattttggttagtttacccat | |
| AT-Kan-F | caggtcgactctagagaaccatttgaggtgatagg | |
| AT-Kan-R | cgcggatccaatataaaatttatattaaatggtactaaaacaattcatccag | |
| M13-F | cgccagggttttcccagtcacgac | |
| M13-R | caggaaacagctatgacc | |
| chro-b’ | gattgaagataggaaatggcag | |
| chro-c’ | gtccggtcgatcagggag | |
| attB-F | gaatgattcccgtccaatgca | |
| attB-R | atacgcaacaggtttggagc | |
| P_int_-F2 | cccgtcgacatttccttggaggttcagcattaacgcttt | |
| P_int_-R2 | tgctctagagaaatgattatgctcccttcaaaat | |
| rpoB-RT-F | tggaagaagcagggatga | |
| rpoB-RT-R | aagtgagtaaggacccgttg | |
| int1-RT-F | catcagtacgcgaaatgaaca | |
| int1-RT-R | tagaggggcttcgtcaggta | |
| int2-RT-F | tacgcagctgctacttttgg | |
| int2-RT-R | ggttgatctctgccttttgg | |
| hp3-RT-F | ccgatctctactgagaatttcg | |
| hp3-RT-R | tattcctctgcgccaattaaa | |

^a^: Sequences of the restriction sites are underlined.

Table S3. Relative copy number of *attI* in wild type, knockout mutants and complemented strains of *L. sphaericus*.

| **Strain** | ***attI*** | | ***adk*** | | **Relative copy number of *attI* (x*_attI_*/x*_adk_*)** |
| --- | --- | --- | --- | --- | --- |
|  | ***Ct*** | Copy number **(x*_attI_*)** | ***Ct*** | Copy number **(x*_adk_*)** |  |
| G725 | 29.91±0.06 | 1.52±0.02 | 12.59±0.12 | 7.62±0.03 | 0.20±0.00 |
| G725△*reg16-17* | 22.41±0.18 | 3.90±0.06 | 12.63±0.08 | 7.60±0.02 | 0.51±0.01 |
| G725△*reg16-17* | 30.34±0.17 | 1.38±0.05 | 12.54±0.31 | 7.63±0.09 | 0.18±0.01 |
| 1593 | 25.22±0.08 | 3.00±0.03 | 12.54±0.19 | 7.63±0.06 | 0.39±0.00 |
| 1593△*int1*_cm1-3 | 21.57±0.34 | 4.17±0.11 | 14.84±0.16 | 6.94±0.05 | 0.60±0.01 |
| 1593△*int2*_cm1-3 | 20.64±0.26 | 4.46±0.08 | 14.70±0.08 | 6.98±0.02 | 0.64±0.01 |
| 1593△*hp3*_cm1-3 | 20.02±0.06 | 4.66±0.02 | 12.79±0.18 | 7.55±0.05 | 0.62±0.00 |
| 1593△*reg16-17* | 21.29±0.12 | 4.26±0.04 | 13.81±0.11 | 7.25±0.03 | 0.59±0.00 |
| 1593△*reg16-17*_cm16 | 30.39±0.17 | 1.36±0.05 | 16.21±0.15 | 6.53±0.04 | 0.21±0.01 |
| 1593△*reg16-17*_cm17 | 26.45±0.19 | 2.62±0.06 | 16.84±0.09 | 6.34±0.03 | 0.41±0.01 |
| 1593△*reg16-17*_cm16-17 | 28.37±0.14 | 2.01±0.05 | 16.85±0.03 | 6.34±0.01 | 0.32±0.01 |


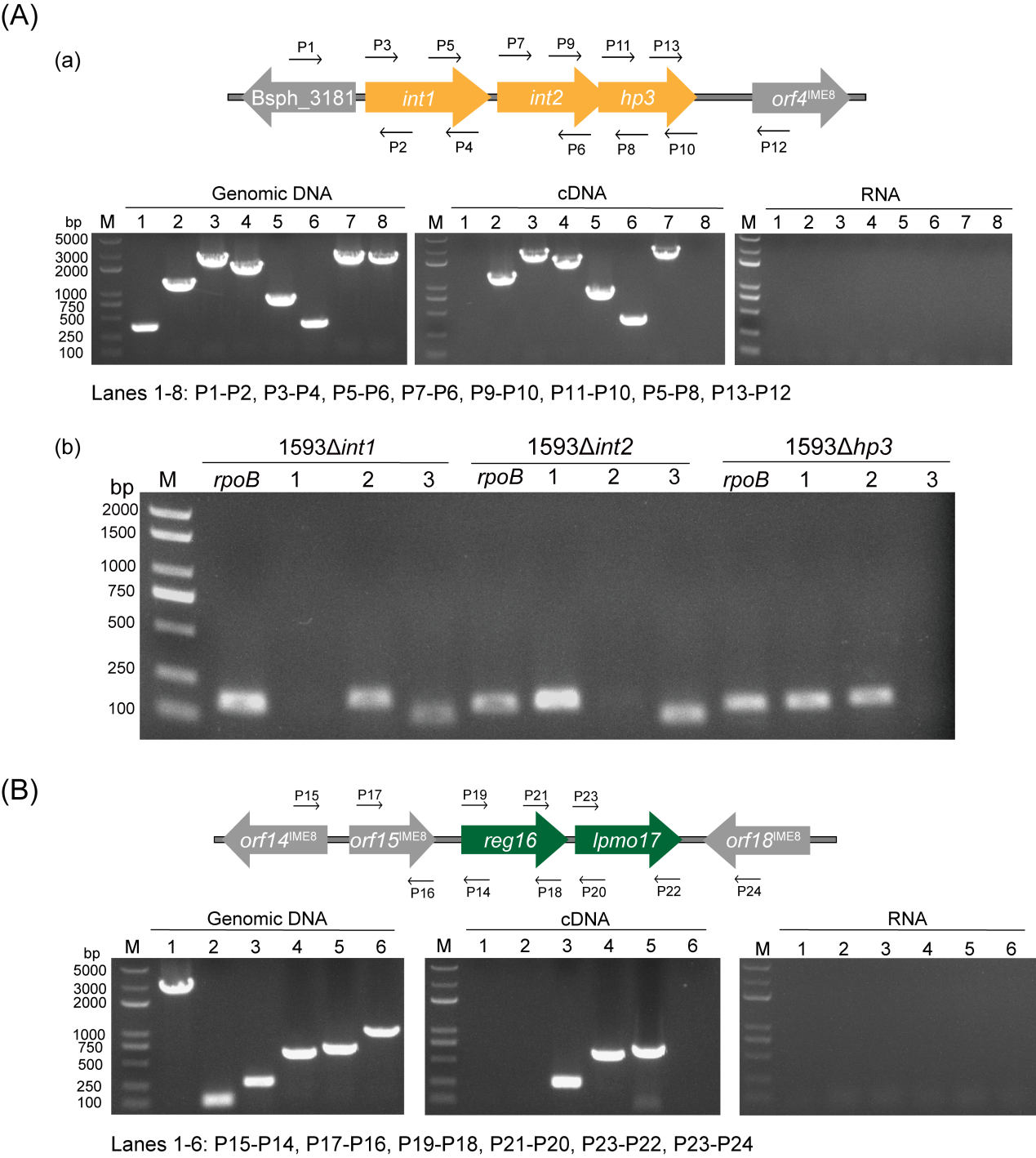


**Figure S1.** Validation of operons *int1-3* (A) and *reg15-17* (B).

(**Aa**) Schematic representation of the primer design (top) and PCR detection (bottom) for *int-*operon validation. Lanes 1-8 refer to primer pairs P1-P2, P3-P4, P5-P6, P7-P6, P9-P10, P11-P10, P5-P8, P13-P12, respectively. Lane M: DL 5000 DNA Marker. The gDNA, total mRNA and cDNA, extracted from C3-41, were used as templates.

(**Ab**) PCR detection of the transcription of *int1*, *int2* and *hp3* in 1593Δ*int1*, 1593Δ*int2* and 1593Δ*hp3* cDNA. “*rpoB*”: reference gene. Lanes 1-3 refer to *int1*, *int2* and *hp3*, respectively. M: DL 2000 DNA Marker.

(**B**) Schematic representation of the primer design (top) and PCR detection (bottom) for *reg15-17* operon validation. Lanes 1-6 refer to primer pairs P15-P14, P17-P16, P19-P18, P21-P20, P23-P22, P23-P24, respectively. Lane M: DL 5000 DNA Marker. The gDNA, total mRNA and cDNA, extracted from C3-41, were used as templates.


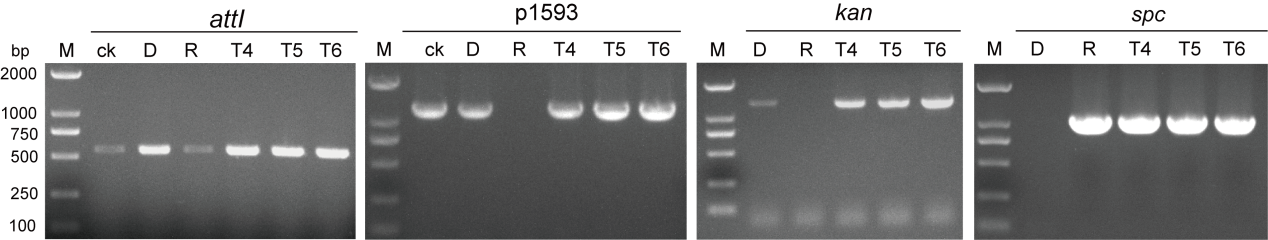


**Figure S2.** PCR detection of the transconjugants carrying circular IME8::Kan^R^ and p1593. The *reg16-17* operon of the IME8 in the donor 1593Δ*reg16-17* was replaced by a kanamycin gene and therefore named IME8::Kan^R^. The primer pair chro-b/c was used for detecting the *attI* site witnessing the circular IME8::Kan^R^ and Tubz-F/R was used for detecting the replicon gene of p1593. “ck”: wild-type strain 1593, “D”: donor strain1593Δ*reg16-17*, “R”: recipient strain G725Δ0498. T4, T5, and T6 transconjugants were randomly picked after the mating experiments. M: DL 2000 DNA Marker.

**References to supporting information.**

1. Poncet S, Bernard C, Dervyn E, Cayley J, Klier A, Rapoport G. 1997. Improvement of *Bacillus sphaericus* toxicity against dipteran larvae by integration, via homologous recombination, of the *Cry11A* toxin gene from *Bacillus thuringiensis* subsp. *israelensis*. Appl Environ Microbiol 63(11):4413-20. https://doi.org/10.1128/aem.63.11.4413-4420.1997.
2. Bourgouin C, Delécluse A, De La Torre F, Szulmajster J. 1990. Transfer of the toxin protein genes of *Bacillus sphaericus* into *Bacillus thuringiensis* subsp. *israelensis* and their expression. Appl Environ Microbiol 56(2): 340-344. https://doi.org/10.1128/aem.56.2.340-344.1990.
3. Fu P, Xiang X, Ge Y, Yuan ZM, Hu XM. 2017. Differential expression of duplicated binary toxin genes *binA/binB* in *Lysinibacillus sphaericus* C3-41. Lett Appl Microbiol 65(1):90-97. https://doi.org/10.1111/lam.12752.
4. Perchat S, Dubois T, Zouhir S, Gominet M, Poncet S, Lemy C, Aumont-Nicaise M, Deutscher J, Gohar M, Nessler S, Lereclus D. 2011. A cell-cell communication system regulates protease production during sporulation in bacteria of the *Bacillus cereus* group. Mol Microbiol 82(3):619-33. https://doi.org/10.1111/j.1365-2958.2011.07839.x.
5. Hu XM, Fan W, Han B, Liu HZ, Zheng DS, Li QB, Dong W, Yan JP, Gao MY, Berry C, Yuan ZM. 2008. Complete genome sequence of the mosquitocidal bacterium *Bacillus sphaericus* C3-41 and comparison with those of closely related *Bacillus* species. J Bacteriol 190(8):2892-2902. https://doi.org/10.1128/JB.01652-07.
6. Ge Y, Zhao N, Hu XM, Shi T, Cai Q, Yuan ZM. 2014. A novel transcriptional activator, *tubX*, is required for the stability of *Bacillus sphaericus* mosquitocidal plasmid pBsph. J Bacteriol 96(24):4304-14. https://doi.org/10.1128/JB.01855-14.
7. Geng PL, Cheng J, Yuan ZM, Xiong H, Wang H, Hu XM. 2010. Horizontal transfer of large plasmid with type IV secretion system and mosquitocidal genomic island with excision and integration capabilities in *Lysinibacillus sphaericus*. Environ Microbiol 23(9):5131-5146. https://doi.org/10.1111/1462-2920.15467.
8. Qin J, Cao Z, Cai X, Fang Y, An B, Li X, Zhang Y, Tian H, Hu W, Yan B, Cai J. 2022. NupR responding to multiple signals is a nucleoside permease regulator in *Bacillus thuringiensis* BMB171. Microbiol Spectr 10(4):e0154322. https://doi.org/10.1128/spectrum.01543-22.
